# Supplementary material for: Enantioselective total synthesis of (‒)-lucidumone enabled by tandem prins cyclization/cycloetherification sequence
Source: Nat Commun. 2024 Mar 26;15:2647. doi: 10.1038/s41467-024-46896-3 (PMC10966040; doi:10.1038/s41467-024-46896-3)
Supplement: Supplementary file 1 — Supplementary Information [file 41467_2024_46896_MOESM1_ESM.pdf]

Supplementary Information

Enantioselective Total Synthesis of (–)-Lucidumone Enabled by Tandem Prins Cyclization/Cycloetherification Sequence

Authors: Xian-Zhang Liao, Ran Wang, Xin Wang, and Guang Li*

Corresponding author. Email: guang.li@imm.ac.cn

Contents

Supplementary Information 1
1. Supplementary Methods 2
2. Supplementary Discussion 3
3. Supplementary Notes 8
    3.1 Detailed experimental procedure 8
    3.2 NMR spectra and HPLC spectra 21
    3.3 Crystallographic information 40
4. Supplementary References 44

1. Supplementary Methods

Unless otherwise stated, reagents were purchased at the highest commercial quality and used without further purification. Solvents were purchased in HPLC quality, degassed by purging thoroughly with nitrogen and dried over activated molecular sieves of appropriate size. Alternatively, they were purged with argon and passed through alumina columns in a solvent purification system (Innovative Technology). Conversion was monitored by thin layer chromatography (TLC) using Merck TLC silica gel 60 F254. Compounds were visualized by UV light at 254 nm and by dipping the plates in an ethanolic vanillin/sulfuric acid solution or an 72 aqueous potassium permanganate solution followed by heating. Flash column chromatography was performed over silica gel (230–400 mesh).

NMR spectra were recorded on a Bruker 500 MHz and 400 MHz at room temperature, Chemical shifts (δ) were reported in parts per million (ppm) relative to residual solvent peaks rounded to the nearest 0.01 for proton and 0.1 for carbon. Coupling constants (J) were reported in Hz to the nearest 0.1 Hz. Peak multiplicity was indicated as follows s (singlet), d (doublet), t (triplet), q (quartet), m (multiplet) and br (broad). Attribution of peaks was done using the multiplicities and integrals of the peaks.

IR spectra were recorded in a Perkin-Elmer 1000 series FT-IR spectrometer. The spectra were reported in cm^{-1}.

The accurate masses were measured by the mass spectrometry service of IMM, PUMC&CAMS on an Agilent 6244 Tof-MS using ESI (Electrospray Ionization).

2. Supplementary Discussion

Supplementary Table 1. Catalytic enantioselective intramolecular Diels-Alder cycloaddition: optimization of reaction conditions.[a]

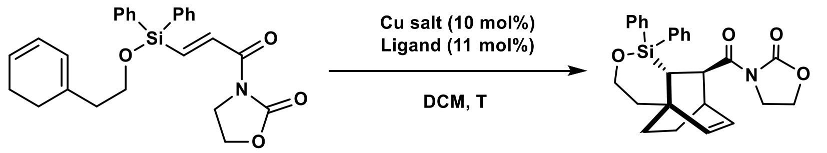

12 11

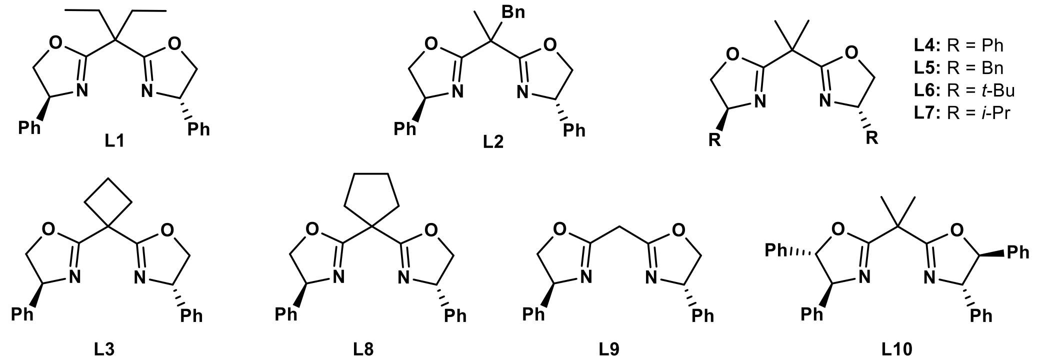

Entry[b]	Catalyst	T[°C]	Yield [%][b]	ee [%][c]
1	**L6**/Cu(OTf)$_2$	r.t	n.r	-
2	**L6**/Cu(SbF$_6$)$_2$	r.t	80	87
3	**L6**/Cu(SbF$_6$)$_2$	50	86	87
4	**L6**/Cu(SbF$_6$)$_2$	70	82	87
5	**L6**/Cu(SbF$_6$)$_2$	90	85	86
6	**L7**/Cu(SbF$_6$)$_2$	50	85	17
7[e]	**L4**/Cu(SbF$_6$)$_2$	50	84	89
8[e]	**L4**/Cu(OTf)$_2$	50	95	91
9[e]	**L5**/Cu(SbF$_6$)$_2$	50	88	60
10[e]	**L1**/Cu(SbF$_6$)$_2$	50	88	92
**11[e]**	**L1/Cu(OTf)$_2$**	**50**	**96**	**92**
12[d][e]	**L1**/Cu(OTf)$_2$	50	95	92
13[e]	**L2**/Cu(OTf)$_2$	50	90	85
14[e]	**L3**/Cu(OTf)$_2$	50	86	63
15[e]	**L8**/Cu(OTf)$_2$	50	85	77
16	**L9**/Cu(OTf)$_2$	50	n.r	-
17	**L10**/Cu(OTf)$_2$	50	n.r	-

[a] Standard conditions: **12** (0.1 mmol), CuX_2 (0.01 mmol), **L*** (0.011 mmol), DCM (*c* 0.1 M), sealed tube, nitrogen. [b] Isolated yields. [c] Determined by HPLC analysis. [d] **12** (3.0 mmol), $Cu(OTf)_2$ (0.3 mmol), **L1** (0.33 mmol), DCM (*c* 0.1 M), sealed tube, nitrogen. [e] Configuration inverse. Abbreviations: DCM = dichloromethane.

Supplementary Table 2. Optimization of Suzuki cross-coupling reaction conditions

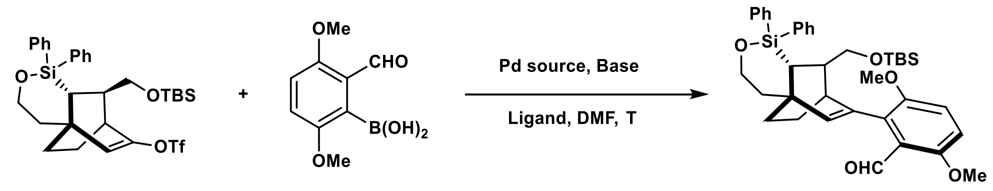

(+)-**10**   **9**   (+)-**8**

Entry	Conditions[a]	Yield of (+)-**8**(%)[b]
**1**	**none**	**69**
2	degassed dioxane instead of degassed DMF	20
3	K_2CO_3 instead of K_3PO_4	42
4	without S-Phos	40
5	$PdCl_2$ instead of $Pd(dppf)Cl_2$	35
6	$Pd(OAc)_2$ instead of $Pd(dppf)Cl_2$	30
7	25°C instead of 80°C	n.r.

[a] All the reactions were performed using (+)-**10** (0.1 mmol), **9** (0.15 mmol), with $Pd(dppf)Cl_2$ (10 mol%) and S-Phos (20 mol%), K_3PO_4 (0.3 mmol) in degassed DMF (2.0 mL) at 80 °C for 3 h under N_2 atmosphere in a Schlenk tube. [b] Isolated yield.

Supplementary Table 3. **Comparison of ^{1}H and ^{13}C NMR data for isolated lucidumone and synthetic lucidumone in CD_3OD**

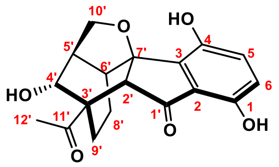

(–)-**lucidumone, 1**

	proton (*J* in Hz)		carbon	
No.	synthetic	isolated	synthetic	isolated
1			149.5, C	149.5, C
2			125.4, C	125.4, C
3			135.4, C	135.4, C
4			150.8, C	150.8, C
5	7.02, d (8.8)	7.01, d (8.7)	126.4, CH	126.4, CH
6	6.76, d (8.7)	6.75, d (8.7)	118.6, CH	118.6, CH
1’			204.3, C	204.2, C
2’	2.89, d (2.3)	2.88, d (2.2)	62.4, CH	62.3, CH
3’			52.7, C	52.7, C
4’	3.92 – 3.89 m	3.90, brs	79.5, CH	79.5, CH
5’	2.36, t-like (4.6)	2.35, t-like (4.5)	48.7, CH	48.7, CH
6'	2.70, q-like (3.3)	2.69, q-like (3.4)	39.1, CH	39.1, CH
7'			87.4, C	87.4, C
8’	Ha 1.92-1.83, m	Ha 1.86, m	16.2, CH_2	16.2, CH_2
	Hb 1.07, dddd	Hb 1.06, m		
9’	Ha 2.21-2.15, m	Ha 2.17, m	15.9, CH_2	15.8, CH2
	Hb 1.38-1.32, m	Hb 1.33, m		
10’	Ha 4.26, dd (8.3, 4.9)	Ha 4.25, dd (8.3, 4.9)	74.3, CH_2	74.3, CH_2
	Hb 3.78, d (8.4)	Hb 3.77, d (8.3)		
11’			214.8, C	214.8, C
12’	2.29, s	2.26, s	28.5, CH_3	28.5, CH_3

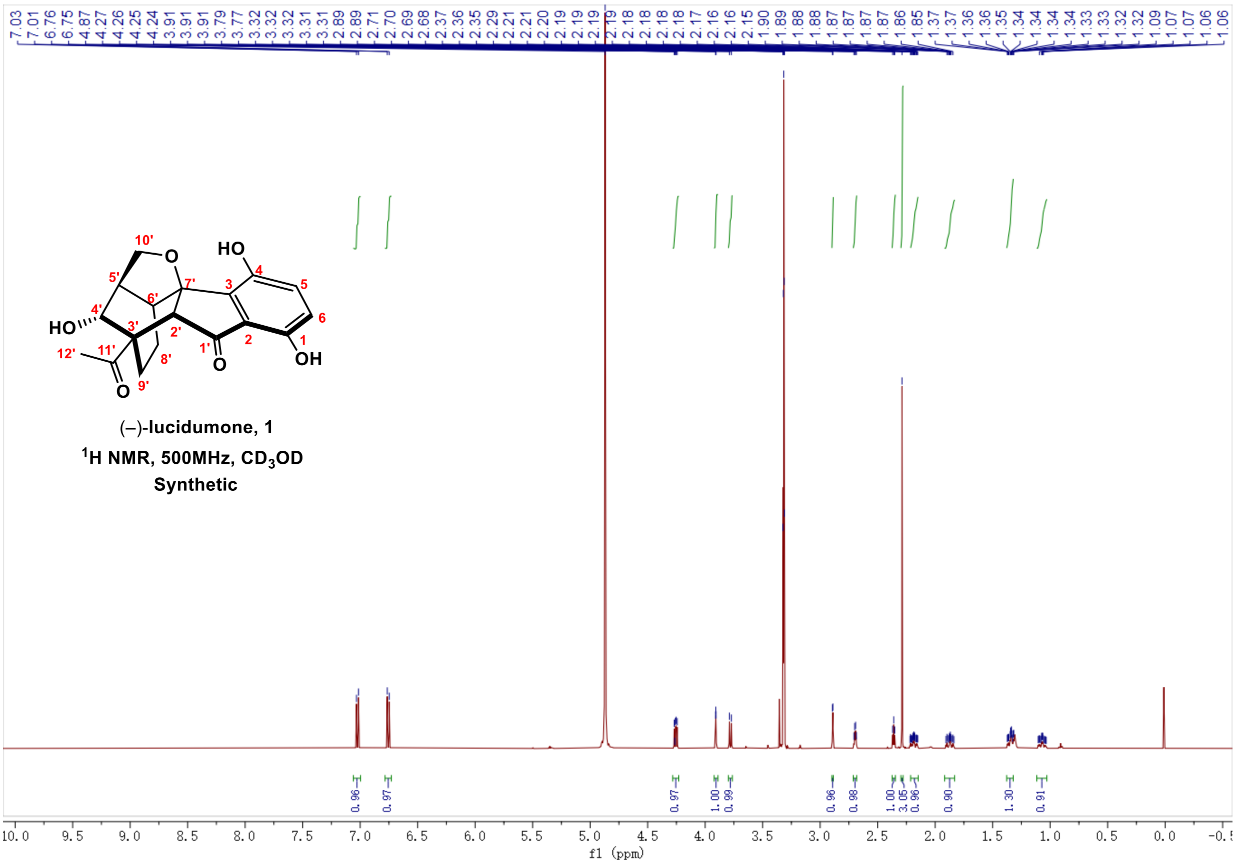

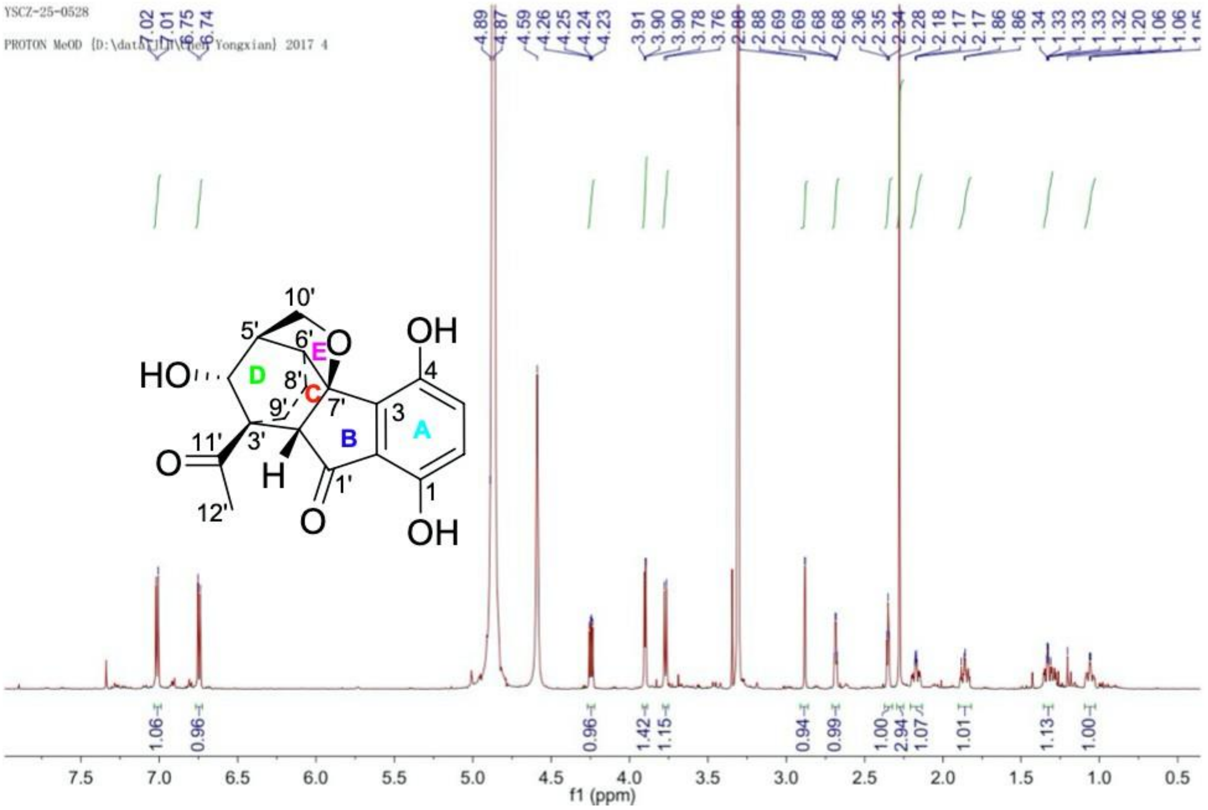

Supplementary Figure 1. ^{1}H NMR Data of synthetic and isolated lucidumone

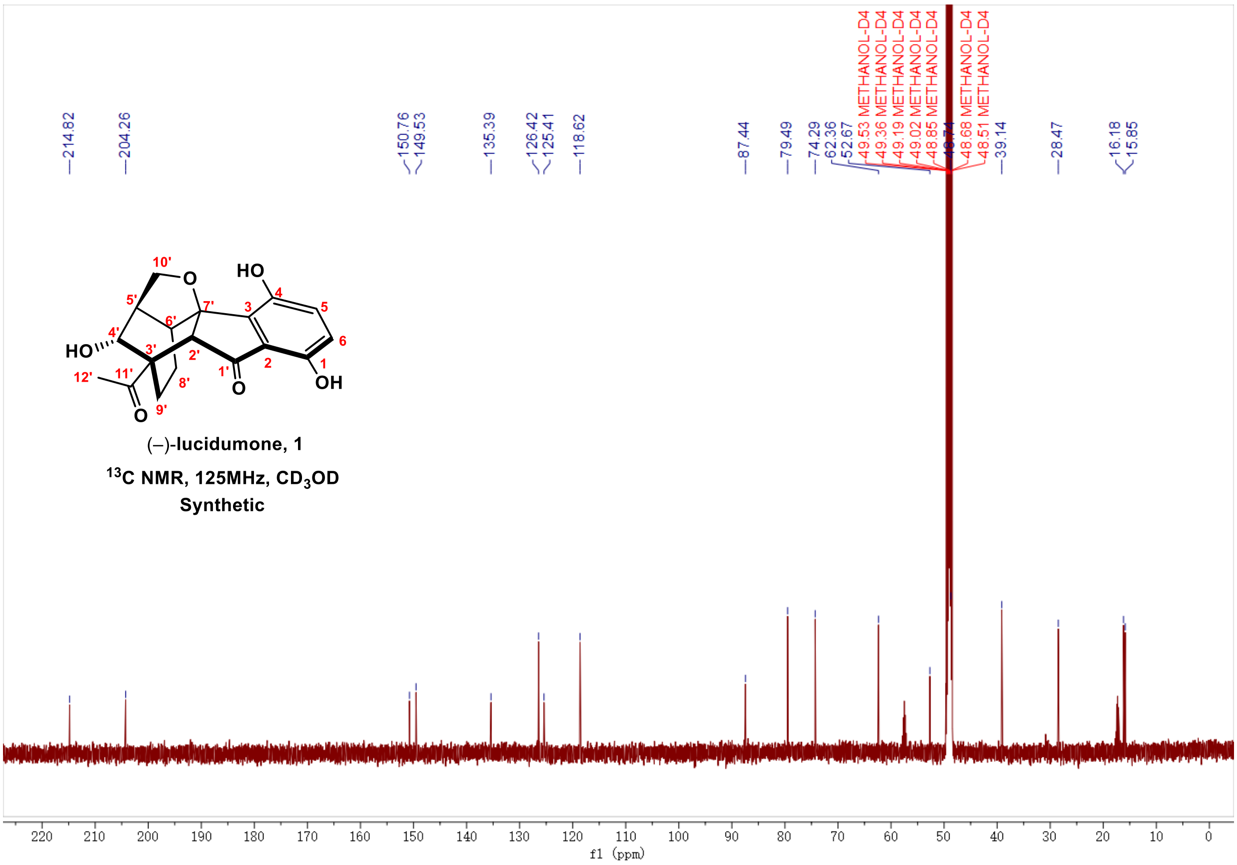

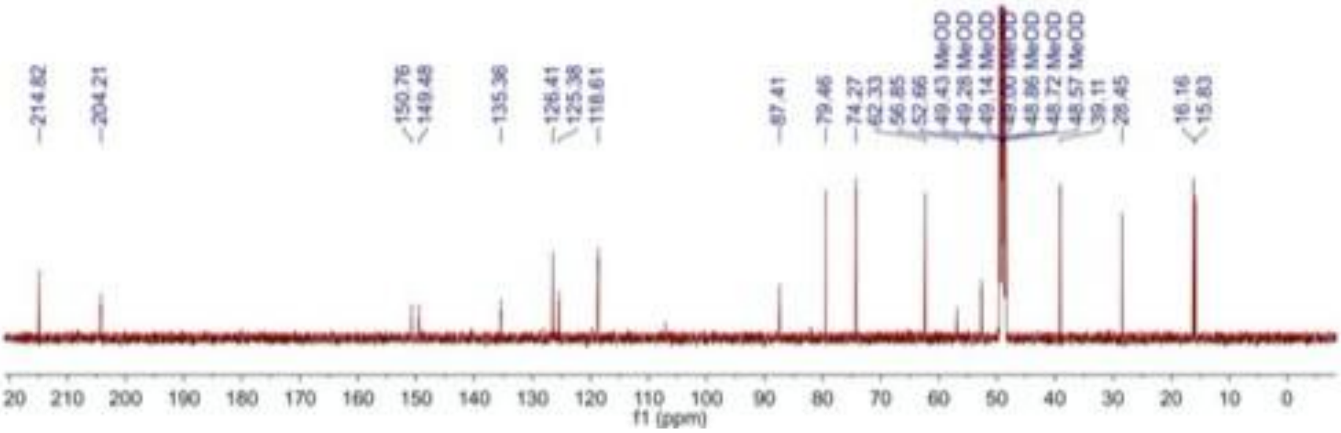

Supplementary Figure 2. ^{13}C NMR Data of synthetic and isolated lucidumone

3. Supplementary Notes

3.1 Detailed experimental procedure

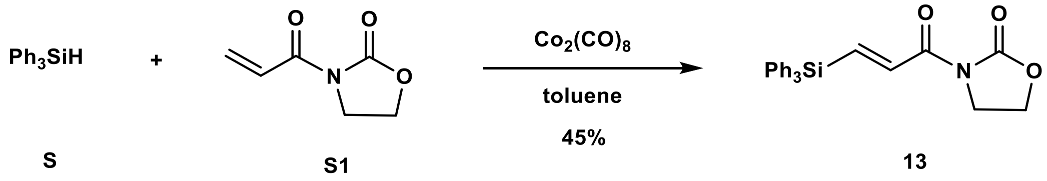

A mixture of triphenysilane **S** (2.60 g, 10 mmol), $Co_2(CO)_8$ (136.0 mg, 0.4 mmol), **S1** (7.05 g, 50 mmol) and 50 mL of degased toluene were added to a 200 mL Schlenk tube unde N_2 atmosphere, and stirred for 12 h at room temprature. Then the reaction mixture was cooled to 0 ℃ and quenched by saturated aqueous NH_4Cl solution, extracted with EtOAc (three times). The combined organic layers were washed with brine, dried over anhydrous Na_2SO_4, filtered, concentrated in *vacuo*. The residue was purified by silica gel flash chromatography (petroleum ether/ethyl acetate = 10/1) to afford compound **13** (1.81 g, 45%) as a white solid[1].

Compound 13

TLC (petroleum ether/ethyl acetate, 5:1 v/v): Rf = 0.3.

^{1}H NMR (400 MHz, $CDCl_3$) δ 7.88 (d, *J* = 18.5 Hz, 1H), 7.68 (d, *J* = 18.5 Hz, 1H), 7.57 – 7.51 (m, 6H), 7.48 – 7.36 (m, 9H), 4.41 (t, *J* = 7.9 Hz, 2H), 4.07 (t, *J* = 8.0 Hz, 2H).

^{13}C NMR (100 MHz, $CDCl_3$) δ 164.6, 153.3, 145.0, 136.9, 136.0, 132.7, 130.1, 128.1, 62.2, 42.7.

IR (KBr): ν (cm^{-1}) 3068, 3048, 2996, 2923, 1781, 1711, 1681, 1477, 1428, 1386, 1361, 1345, 1270, 1223, 1112, 1026, 998, 964, 844, 758, 741, 709, 700, 653, 617.

HRMS (ESI):*m/z* calcd. for $C_{24}H_{22}NO_3Si$ $[M+H]^+$: 400.1369, found 400.1364.

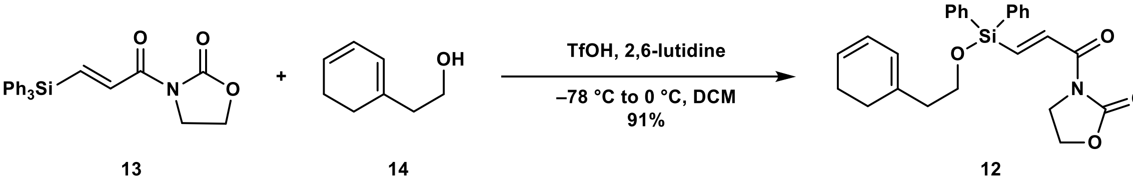

TfOH (790 μL, 8.9 mmol) was added to a solution of **13** (1.62 g, 4.1 mmol) in DCM (50 mL) at 0 ºC under N_2 atmosphere and stirred for 3 h. The mixture was cooled to **–**78 ºC and 2,6-lutidine (1.2 mL, 9.8 mmol) was added dropwise. After stirring for another 5 minutes, **14**[2] (554.7 mg, 4.5 mmol) was added. Then the mixture was warmed to 0 ºC, quenched by saturated aqueous NH_4Cl solution (100 mL) and extracted with DCM (three times). The combined organic layers were washed with brine, dried over anhydrous Na_2SO_4, and concentrated *in vacuo*. The residue was subjected to flash chromatography on silica gel (petroleum ether/ethyl acetate = 10:1 to 5:1) to afford **12** (1.63 g, 91%) as a colorless oil[3].

Compound 12

TLC (petroleum ether/ethyl acetate, 5:1 v/v): Rf = 0.2.

^{1}H NMR (400 MHz, $CDCl_3$) δ 7.82 (d, *J* = 18.6 Hz, 1H), 7.67 – 7.58 (m, 5H), 7.46 – 7.36 (m, 6H), 5.90 – 5.81 (m, 1H), 5.66 (m, 2H), 4.42 (t, *J* = 8.0 Hz, 2H), 4.08 (t, *J* = 8.0 Hz, 2H), 3.88 (t, *J* = 7.0 Hz, 2H), 2.38 (t, *J* = 7.0 Hz, 2H), 2.16 – 2.08 (m, 2H), 2.08 – 1.99 (m, 2H).

^{13}C NMR (100 MHz, $CDCl_3$) δ 164.5, 153.3, 144.0, 136.4, 136.0, 135.0, 132.9, 130.5, 128.1, 124.6, 124.0, 120.6, 62.8, 62.2, 42.7, 40.4, 26.5, 22.8.

IR (KBr): ν (cm^{-1}) 2956, 2923, 2853, 1781, 1743, 1682, 1590, 1466, 1429, 1385, 1362, 1344, 1270, 1212, 1191, 1112, 1082, 1052, 1032, 969, 867, 847, 829, 758, 741, 715, 700, 662.

HRMS (ESI):*m/z* calcd. for $C_{26}H_{28}NO_4Si$ $[M+H]^+$: 446.1788, found 446.1782.

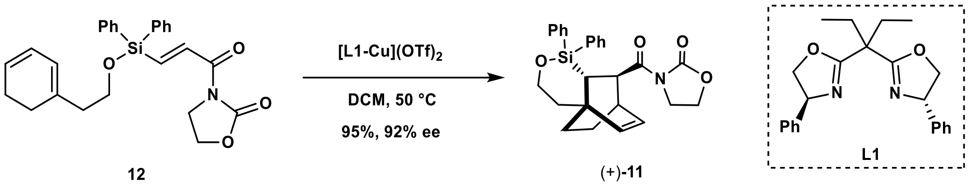

2

Cu(OTf)$_2$ (109.5 mg, 0.3 mmol) was added to a solution of (4*S*,4'*S*)- 2,2'-(1-ethylpropylidene)bis[4,5-dihydro-4-phenyl-oxazole] (**L1**, 120.1 mg, 0.33 mmol) in DCM (20 mL) under N_2 atmosphere and stirred for 3 h to deliver [**L1**–Cu](OTf)$_2$ complex which was used without further purification. Subsequently, compound **12** (3.0 mmol, 1.32 g) in 20 mL of DCM was added to [**L1**-Cu](OTf)$_2$ (in 20 mL of DCM) under N_2 atmosphere and stirred at 50 °C for 12 h. Upon completion, the reaction was quenched by saturated aqueous NH_4Cl solution and extracted with DCM (three times). The combined organic layers were washed with brine, dried over anhydrous Na_2SO_4, and concentrated. The residue was subjected to flash chromatography on silica gel (petroleum ether/ethyl acetate = 10:1 to 5:1) to afford (+)-**11** (1.25 g, 95%, 92% ee) as a colorless oil.

Compound (+)-11

TLC (petroleum ether/ethyl acetate, 5:1 v/v): Rf = 0.2.

Optical rotation: $[\alpha]_D^{20}$ = +42 (c = 1.0, MeOH).

^{1}H NMR (400 MHz, $CDCl_3$) δ 7.74 (dt, *J* = 4.6, 2.6 Hz, 2H), 7.46 (tt, *J* = 3.3, 1.8 Hz, 5H), 7.38 – 7.26 (m, 3H), 6.24 (d, *J* = 8.2 Hz, 1H), 6.00 (dd, *J* = 8.3, 6.4 Hz, 1H), 4.42 (td, *J* = 13.1, 12.3, 2.3 Hz, 1H), 4.34 (td, *J* = 9.0, 7.3 Hz, 1H), 4.31 – 4.16 (m, 3H), 3.88 (dddd, *J* = 30.4, 11.1, 9.2, 6.9 Hz, 2H), 2.83 (dq, *J* = 5.7, 3.4 Hz, 1H), 2.39 (td, *J* = 13.6, 13.1, 5.0 Hz, 1H), 2.09 (dd, *J* = 7.3, 3.1 Hz, 1H), 1.88 – 1.76 (m, 2H), 1.35 – 1.26 (m, 1H), 1.19 (tt, *J* = 12.3, 3.9 Hz, 1H), 0.84 (tq, *J* = 12.1, 4.5, 3.8 Hz, 1H).

^{13}C NMR (100 MHz, $CDCl_3$) δ 174.8, 153.2, 142.8, 135.3, 134.8, 134.6, 134.1, 130.2, 130.0, 128.4, 128.1, 127.8, 61.8, 61.6, 43.0, 42.5, 38.8, 36.9, 35.1, 28.7, 26.3, 25.8.

IR (KBr): ν (cm^{-1}) 2924, 2868, 1775, 1697, 1478, 1427, 1385, 1361, 1317, 1277, 1255, 1193, 1108, 1075, 1040, 999, 982, 956, 916,857, 759, 738, 709, 701, 686, 664.

HRMS (ESI):*m/z* calcd. for $C_{26}H_{28}NO_4Si$ $[M+H]^+$: 446.1788, found 446.1771.

Chiral HPLC: Chiralpak OD-H, hexane:*i*-PrOH = 80:20, 1.0 mL/min, 210 nm; tR = 11.8 min (minor), 15.9 min (major).

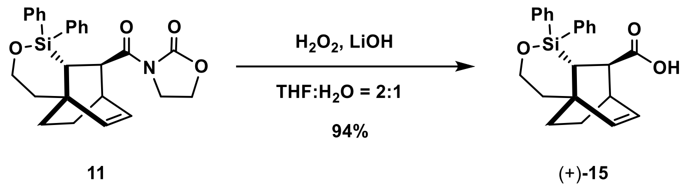

To a solution of compound **11** (448.6 mg, 1.0 mmol) in THF (10 mL) and H_2O (5.0 mL) was added 30 wt.% H_2O_2 in H_2O (1.0 mL, 8.8 mmol), LiOH (72.2 mg, 3.0 mmol) at 0 °C. The mixture was stirred for 5 h then quenched by saturated $Na_2S_2O_3$ solution. The pH was adjusted to 1 by addition of 1M HCl and extracted with EtOAc (three times). The combined organic layers were washed with brine, dried over anhydrous Na_2SO_4, filtered and concentrated in *vacuo*. The resulting residue was purified by silica gel column chromatography (petroleum ether/ethyl acetate = 10:1) to afford (+)-**15** (354.2 mg, 94%) as white solid.

Compound (+)-15

TLC (petroleum ether/ethyl acetate, 5:1 v/v): Rf = 0.4.

Optical rotation: $[\alpha]_D^{20}$ = +10 (c = 1.0, MeOH).

^{1}H NMR (500 MHz, $CDCl_3$) δ 7.73 – 7.69 (m, 2H), 7.57 – 7.54 (m, 2H), 7.47 – 7.41 (m, 3H), 7.37 – 7.33 (m, 1H), 7.30 – 7.26 (m, 2H), 6.20 (dt, *J* = 8.2, 1.2 Hz, 1H), 6.14 (dd, *J* = 8.2, 6.3 Hz, 1H), 4.45 (ddd, *J* = 13.0, 11.5, 2.6 Hz, 1H), 4.23 (ddd, *J* = 11.5, 5.2, 1.7 Hz, 1H), 2.94 – 2.88 (m, 1H), 2.81 (dd, *J* = 7.6, 2.0 Hz, 1H), 2.31 (ddd, *J* = 14.3, 13.0, 5.3 Hz, 1H), 1.88 (ddd, *J* = 12.1, 9.6, 4.3 Hz, 1H), 1.81 (ddd, *J* = 14.3, 2.6, 1.7 Hz, 1H), 1.76 (dd, *J* = 7.6, 3.0 Hz, 1H), 1.24 – 1.17 (m, 1H), 1.07 (dddd, *J* = 12.4, 9.9, 4.3, 2.4 Hz, 1H), 0.95 – 0.87 (m, 1H).

^{13}C NMR (125 MHz, $CDCl_3$) δ 179.9, 142.5, 135.6, 134.82, 134.78, 134.4, 130.2, 130.0, 129.9, 128.0, 127.9, 61.8, 44.9, 38.5, 36.8, 34.2, 30.5, 26.3, 25.7.

IR (KBr): ν (cm^{-1}) 3346, 2971, 2932, 2883, 1467, 1409, 1379, 1340, 1310, 1161, 1129, 1109, 953, 817.

HRMS (ESI):*m/z* calcd. for $C_{23}H_{25}O_3Si$ $[M+H]^+$: 377.1573, found 377.1568.

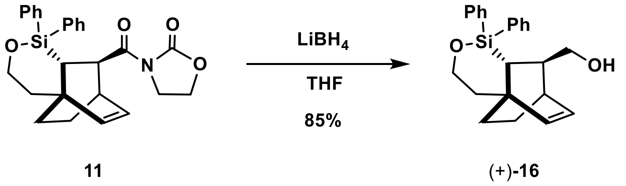

To a solution of compound **11** (44.2 mg, 0.1 mmol) in THF (2.0 mL) was added $LiBH_4$ (1.1 mmol, 23.6 mg) at 0 °C. The mixture was stirred for 3 h at r.t., then quenched by saturated NH_4Cl solution and extracted with EtOAc (three times). The combined organic layers were washed with brine, dried over anhydrous Na_2SO_4, filtered and concentrated in *vacuo*. The resulting residue was purified by silica gel column chromatography (petroleum ether/ethyl acetate = 7:1) to afford (+)-**16** (30.5 mg, 85%) as colorless oil.

Compound (+)-16

TLC (petroleum ether/ethyl acetate, 4:1 v/v): Rf = 0.4.

Optical rotation: $[\alpha]_D^{20}$ = +15 (c = 1.0, MeOH).

¹H NMR (400 MHz, $CDCl_3$) δ 7.85 – 7.77 (m, 2H), 7.58 – 7.52 (m, 2H), 7.46 (td, *J* = 4.4, 3.9, 2.3 Hz, 3H), 7.34 (ddd, *J* = 14.2, 7.7, 6.0 Hz, 3H), 6.16 – 6.08 (m, 2H), 4.41 (ddd, *J* = 13.3, 11.6, 2.4 Hz, 1H), 4.19 (ddd, *J* = 11.6, 5.1, 1.9 Hz, 1H), 3.11 (dd, *J* = 6.9, 2.9 Hz, 2H), 2.64 (dq, *J* = 5.4, 2.6 Hz, 1H), 2.32 – 2.16 (m, 2H), 1.79 (tdd, *J* = 14.2, 7.1, 3.3 Hz, 2H), 1.20 (dt, *J* = 12.1, 4.0 Hz, 1H), 1.11 (dddd, *J* = 12.3, 9.8, 4.2, 2.5 Hz, 1H), 0.87 (dt, *J* = 12.1, 3.5 Hz, 1H), 0.73 (dd, *J* = 7.3, 3.1 Hz, 1H).

¹³C NMR (125 MHz, $CDCl_3$) δ 141.9, 136.0, 135.0, 134.7, 134.3, 130.5, 130.3, 129.9, 128.1, 128.0, 67.3, 61.6, 43.0, 39.0, 37.0, 32.5, 32.3, 26.48, 26.46.

IR (KBr): ν (cm^{-1}) 3420, 2922, 2866, 1427, 1263, 1108, 1070, 1032, 911, 859, 804, 773, 755, 734, 698, 677.

HRMS (ESI):*m/z* calcd. for $C_{23}H_{27}O_2Si$ $[M+H]^+$: 363.1780, found 363.1767.

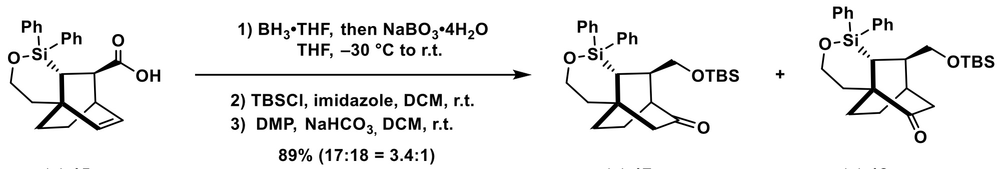

(+)-**15** (+)-**17** (+)-**18**

BH_3•THF (1M in THF, 5.0 mL, 5.0 mmol) was added to a solution of (+)-**15** (376.5 mg, 1.0 mmol) in THF (10 mL) at –30 ºC under N_2 atmosphere. Upon consumption of the starting material as indicated by TLC, the mixture was allowed to warm to room temperature, and stirred for another 12 h at the same temperature. Then the reaction mixture was cooled to 0 ℃ followed by the addition of $NaBO_3$•$4H_2O$ (1.53 g, 10 mmol). The reaction mixture was quenched by saturated aq. NH_4Cl and extracted with EtOAc (three times). The combined organic layers were washed with brine, dried over anhydrous Na_2SO_4, and concentrated in *vacuo* to give crude diol which was used for the next step without further purification.

To a solution of crude diol, imidazole (340 mg, 5.0 mmol) in DCM (20 mL) at room temperature was added TBSCl (453.6 mg, 3.0 mmol). The reaction was completed in 5 h, then $NaHCO_3$ (840mg, 10 mmol) and Dess-Martin periodinane (830.2 mg, 2.0 mmol) was added at 0 °C. The mixture was warmed to room temperature, and the reaction was completed in 2 h, quenched by sat. $Na_2S_2O_3$ aq. and extracted with DCM (three times). The combined organic layers were washed with brine, dried over anhydrous Na_2SO_4 and concentrated in *vacuo*. The resulting residue was purified by silica gel column chromatography (petroleum ether/ethyl acetate = 20:1 to 10:1) to afford (+)-**17** (340.2 mg, 69%) and (+)-**18** (98.6 mg, 20%) both in the form of colorless solid.

Compound (+)-17 (major)

TLC (petroleum ether/ethyl acetate, 10:1 v/v): Rf = 0.35.

Optical rotation: $[\alpha]_D^{20}$ = +53 (c = 1.0, MeOH).

¹H NMR (500 MHz, $CDCl_3$) δ 7.73 – 7.69 (m, 2H), 7.60 – 7.56 (m, 2H), 7.50 – 7.42 (m, 3H), 7.42 – 7.39 (m, 1H), 7.37 – 7.33 (m, 2H), 4.37 (ddd, *J* = 12.9, 11.6, 2.4 Hz, 1H), 4.17 (ddd, *J* = 11.6, 5.1, 1.8 Hz, 1H), 3.44 (dd, *J* = 10.0, 2.7 Hz, 1H), 3.28 (dd, *J* = 10.0, 5.1 Hz, 1H), 2.29 – 2.24 (m, 2H), 2.10 (d, *J* = 1.5 Hz, 2H), 2.01 – 1.93 (m, 2H), 1.71 – 1.62 (m, 1H), 1.55 (dd, *J* = 8.4, 2.9 Hz,

1H), 1.52 (dt, J = 14.3, 2.2 Hz, 1H), 1.47 – 1.41 (m, 1H), 1.21 (dddd, J = 13.3, 11.7, 4.3, 3.0 Hz, 1H), 0.85 (s, 9H), –0.059 (s, 3H), –0.065 (s, 3H).

^{13}C NMR (125 MHz, $CDCl_3$) δ 214.7, 135.6, 135.1, 134.8, 134.0, 130.5, 130.1, 128.14, 128.08, 65.9, 61.4, 55.0, 46.9, 41.2, 40.4, 36.6, 28.9, 26.4, 26.0, 23.8, 18.5, –5.70, –5.73.

IR (KBr): ν (cm^{-1}) 2955, 2927, 2855, 1728, 1470, 1428, 1258, 1180, 1108, 1064, 1018, 912, 892, 878, 861, 834, 801, 734, 705, 685.

HRMS (ESI):*m/z* calcd. for $C_{29}H_{41}O_3Si_2$ $[M+H]^+$: 493.2594, found 493.2582.

Compound (+)-18 (minor)

TLC (petroleum ether/ethyl acetate, 10:1 v/v): Rf = 0.5.

Optical rotation: $[\alpha]_D^{20}$ = +50 (c = 1.0, MeOH).

^{1}H NMR (500 MHz, $CDCl_3$) δ 7.76 – 7.72 (m, 2H), 7.56 – 7.53 (m, 2H), 7.51 – 7.39 (m, 4H), 7.36 – 7.33 (m, 2H), 4.30 (ddd, J = 12.6, 11.5, 2.5 Hz, 1H), 4.23 (ddd, J = 11.4, 5.3, 2.4 Hz, 1H), 3.38 (dd, J = 10.4, 4.2 Hz, 1H), 3.30 (dd, J = 10.4, 8.2 Hz, 1H), 2.69 (dt, J = 19.0, 2.8 Hz, 1H), 2.21 (hept, J = 2.7 Hz, 1H), 2.19 – 2.02 (m, 4H), 1.68 (dt, J = 15.0, 2.4 Hz, 1H), 1.55 – 1.49 (m, 1H), 1.45 – 1.35 (m, 2H), 1.30 (dd, J = 8.9, 2.5 Hz, 1H), 0.82 (s, 9H), -0.11 (d, J = 0.9 Hz, 6H).

^{13}C NMR (125 MHz, $CDCl_3$) δ 215.3, 135.5, 135.1, 134.6, 134.0, 130.5, 130.2, 128.2, 128.1, 65.2, 61.3, 44.7, 39.7, 39.0, 32.5, 29.7, 26.5, 26.0, 26.0, 25.1, 18.3, –5.51, –5.52.

IR (KBr): ν (cm^{-1}) 2928, 2856, 1715, 1470, 1428, 1251, 1180, 1111, 1090, 946, 885, 855, 835, 755, 737, 725, 706, 666, 640, 626.

HRMS (ESI):m/z calcd. for $C_{29}H_{41}O_3Si_2$ [M+H]+: 493.2594, found 493.2581.

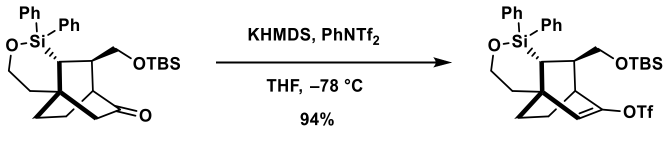

(+)-**17** (+)-**10**

KHMDS (1M in THF, 0.84 mL, 0.84 mmol) was added to a solution of (+)-**17** (300.2 mg, 0.6 mmol) in THF (8.0 mL) at –78 ℃ under N_2 atmosphere. After stirring for 15 minutes at this temperature to ensure full deprotonation, $PhNTf_2$ (300.0 mg, 0.84 mmol) in THF (2.0 mL) was added. The reaction mixture was stirred at the same temperature until TLC indicating full consumption of (+)-**17** (ca. 30 minutes) and then quenched by sat. NH_4Cl solution. The aqueous layer was extracted with EtOAc (three times). The combined extracts were dried over anhydrous Na_2SO_4, filtered, and concentrated in *vacuo*. The resulting residue was purified by silica gel column chromatography (petroleum ether/ethyl acetate = 30:1) to afford (+)-**10** (350.8 mg, 94%) as a colorless solid.

Compound (+)-10

TLC (petroleum ether/ethyl acetate, 30:1 v/v): Rf = 0.30

Optical rotation: $[\alpha]_D^{20}$ = +53 (c = 1.0, MeOH).

^{1}H NMR (500 MHz, $CDCl_3$) δ 7.78 – 7.73 (m, 2H), 7.54 – 7.50 (m, 2H), 7.50 – 7.44 (m, 3H), 7.42 – 7.37 (m, 1H), 7.36 – 7.30 (m, 2H), 5.84 (d, *J* = 2.5 Hz, 1H), 4.41 – 4.33 (m, 1H), 4.20 (ddd, *J* = 11.8, 5.0, 1.9 Hz, 1H), 3.15 (dd, *J* = 10.2, 4.0 Hz, 1H), 3.03 – 2.96 (m, 1H), 2.92 (p, *J* = 2.4 Hz, 1H), 2.35 – 2.21 (m, 2H), 1.86 (dt, *J* = 14.3, 2.2 Hz, 1H), 1.82 – 1.75 (m, 1H), 1.54 (tt, *J* = 12.2, 3.7 Hz, 1H), 1.15 (tdd, *J* = 9.8, 4.3, 2.5 Hz, 1H), 0.97 (tt, *J* = 12.2, 3.8 Hz, 1H), 0.82 (s, 9H), 0.72 (dd, *J* = 7.2, 3.1 Hz, 1H), –0.09 (s, 6H).

^{13}C NMR (125 MHz, $CDCl_3$) δ 149.7, 135.2, 134.7, 134.5, 133.7, 130.4, 130.1, 128.1, 128.0, 126.1, 118.6(q, $J_{C\text{-}F}$ = 318.7 Hz), 65.8, 61.3, 43.8, 40.2, 38.9, 37.2, 31.1, 26.8, 26.2, 25.8, 18.3, –5.69, –5.71.

IR (KBr): v (cm^{-1}) 2963, 2923, 2854, 1650, 1470, 1420, 1262, 1246, 1211, 1141, 1107, 1066, 1032, 903, 887, 836, 805, 777, 760, 732, 706, 670, 646, 606.

HRMS (ESI):*m/z* calcd. for $C_{30}H_{40}F_3O_5SSi_2$ $[M+H]^+$: 625.2087, found 625.2072.

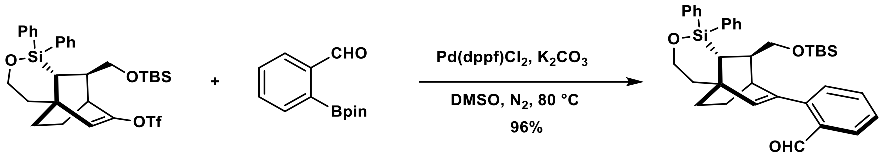

(+)-**10** 19 (+)-**20**

To a sealed tube were added Pd(dppf)Cl_2 (3.9 mg, 0.005 mmol), K_2CO_3 (41.6 mg, 0.3 mmol), **19** (34.8 mg, 0.15 mmol), (+)-**10** (61.9 mg, 0.1 mmol) and DMSO (2.0 mL) under N_2 atmosphere. The resulting mixture was heated to 80 ℃ and stirred for 3 h. Then the reaction was cooled to 0 ℃ and quenched by water, extracted with EtOAc (three times). The combined organic layers were washed with brine, dried over anhydrous Na_2SO_4, filtered, concentrated in *vacuo*. The residue was purified by silica gel flash chromatography (petroleum ether/ethyl acetate = 10/1) to afford compound (+)-**20** (55.3 mg, 96%) as a yellow oil.

Compound (+)-20

TLC (petroleum ether/ethyl acetate, 5:1 v/v): Rf = 0.30.

Optical rotation: $[\alpha]_D^{20}$ = +65 (c = 1.0, MeOH).

^{1}H NMR (400 MHz, CD_3OD) δ 10.10 (s, 1H), 7.88 – 7.82 (m, 3H), 7.70 (dd, *J* = 7.9, 1.1 Hz, 1H), 7.59 – 7.49 (m, 7H), 7.43 – 7.39 (m, 1H), 7.38 – 7.35 (m, 2H), 6.01 (d, *J* = 1.8 Hz, 1H), 4.50 – 4.40 (m, 1H), 4.19 (ddd, *J* = 11.7, 5.0, 1.9 Hz, 1H), 3.24 (q, *J* = 2.4 Hz, 1H), 3.15 – 3.07 (m, 1H), 2.93 (t, *J* = 10.5 Hz, 1H), 2.41 – 2.30 (m, 2H), 1.97 – 1.86 (m, 2H), 1.46 – 1.41 (m, 1H), 1.28 (dt, *J* = 10.0, 2.4 Hz, 1H), 1.07 – 0.99 (m, 1H), 0.80 (s, 9H), 0.66 (dd, *J* = 7.3, 2.9 Hz, 1H), –0.13 (s, 3H), –0.18 (s, 3H).

^{13}C NMR (100 MHz, CD_3OD) δ 192.3, 145.1, 144.5, 138.1, 135.5, 134.5, 134.4, 134.2, 133.8, 133.1, 130.1, 130.0, 128.7, 127.9, 127.6, 127.3, 126.6, 65.9, 61.2, 42.8, 38.53, 38.49, 37.6, 31.2, 26.2, 25.5, 25.0, 17.6, -6.6, -6.8.

IR (KBr): v (cm^{-1}) 2953, 2927, 2854, 1690, 1595, 1487, 1428, 1386, 1253, 1195, 1108, 1092, 1074, 1005, 962, 923, 847, 837, 775, 764, 731, 705, 682, 640.

HRMS (ESI):*m/z* calcd. for $C_{36}H_{44}NaSi_2$ $[M+Na]^+$: 603.2721, found 603.2725.

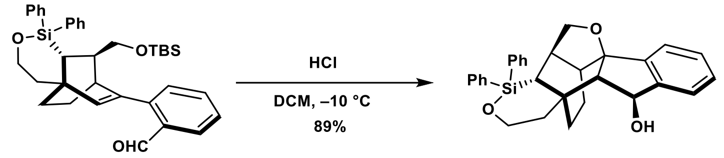

(+)-20 (–)-21

HCl (2M in EtOAc, 0.43 mL, 0.86 mmol) was added to a solution of (+)-**20** (50.7 mg, 0.086 mmol) in DCM (2.0 mL) at –78 ℃ under N_2 atmosphere. After stirring for 12 h at this temperature, the reaction was quenched by H_2O. The aqueous layer was extracted with DCM (three times). The combined extracts were dried over Na_2SO_4, filtered, and concentrated in *vacuo*. The residue was purified by silica gel flash chromatography (petroleum ether/ethyl acetate = 3/1) to afford compound (–)-**21** (41 mg, 89%) as a white solid.

Compound (–)-21

TLC (petroleum ether/ethyl acetate, 2:1 v/v): Rf = 0.33.

Optical rotation: $[\alpha]_D^{20}$ = –31 (c = 1.0, MeOH).

^{1}H NMR (400 MHz, $CDCl_3$) δ 7.78 – 7.72 (m, 2H), 7.58 (dd, *J* = 7.8, 1.8 Hz, 2H), 7.47 – 7.39 (m, 7H), 7.35 (d, *J* = 6.5 Hz, 3H), 4.76 (dd, *J* = 10.5, 5.9 Hz, 1H), 4.31 (td, *J* = 12.1, 2.7 Hz, 1H), 4.18 (ddd, *J* = 11.6, 5.1, 1.9 Hz, 1H), 4.01 (dd, *J* = 7.3, 3.2 Hz, 1H), 3.49 (d, *J* = 7.2 Hz, 1H), 2.98 (d, *J* = 10.5 Hz, 1H), 2.69 (q, *J* = 3.4 Hz, 1H), 1.95 (q, *J* = 3.6 Hz, 1H), 1.89 (dt, *J* = 14.5, 2.4 Hz, 1H), 1.84 – 1.67 (m, 2H), 1.49 (d, *J* = 5.9 Hz, 1H), 1.24 – 1.13 (m, 3H), 0.85 – 0.74 (m, 1H).

^{13}C NMR (100 MHz, $CDCl_3$) δ 145.5, 140.9, 135.8, 135.3, 134.53, 134.49, 130.3, 129.9, 129.1, 128.9, 128.1,128.1, 124.6, 124.2, 90.0, 76.6, 73.6, 71.6, 61.7, 41.5, 39.9, 36.7, 36.4, 32.7, 21.7, 15.5.

IR (KBr): ν (cm^{-1}) 3391, 2923, 1428, 1112, 1053, 1042, 1032, 1016, 996, 912, 853, 816, 758, 740, 704, 680, 670, 643.

HRMS (ESI):*m/z* calcd. for $C_{30}H_{30}O_3NaSi$ $[M+Na]^+$: 489.1856, found 489.1860.

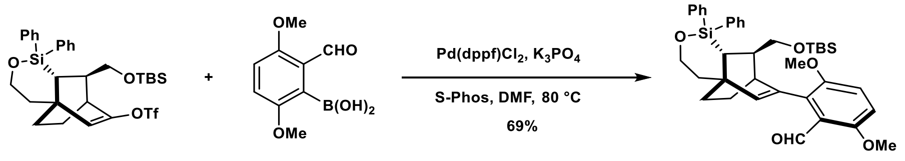

(+)-10 9 (+)-8

To a sealed tube were added Pd(dppf)Cl_2 (36.5 mg, 0.05 mmol), K_3PO_4 (0.97 g, 1.5 mmol), 2-dicyclohexylphosphino-2',6'-dimethoxybiphenyl (S-Phos, 41.2 mg, 0.1 mmol), **9** (213.2 mg, 1.0 mmol) and (+)-**10** (313.3 mg, 0.5 mmol). Degased DMF (10 mL) were then added under N_2 atmosphere. The resulting mixture was heated to 80 ℃ and stirred for 1.5 h. Then the reaction was cooled to 0 ℃ and quenched by sat. $NaHCO_3$ solution, extracted with EtOAc (three times). The combined organic layers were washed with brine, dried over anhydrous Na_2SO_4, filtered, concentrated in *vacuo*. The residue was purified by silica gel flash chromatography (petroleum ether/ethyl acetate/Et_3N = 100/10/1) to afford compound (+)-**8** (219.1 mg, 69%) as a yellow oil.

Compound (+)-8

TLC (petroleum ether/ethyl acetate, 4:1 v/v): Rf = 0.32.

Optical rotation: $[\alpha]_D^{20}$ = +38 (c = 1.0, MeOH).

^{1}H NMR (400 MHz, CD_3OD) δ 10.19 (s, 1H), 7.83 – 7.79 (m, 2H), 7.57 – 7.53 (m, 2H), 7.52 – 7.47 (m, 3H), 7.42 – 7.38 (m, 1H), 7.37 – 7.32 (m, 2H), 7.14 (d, *J* = 9.2 Hz, 1H), 6.98 (d, *J* = 9.1 Hz, 1H), 5.95 (d, *J* = 1.6 Hz, 1H), 4.42 (ddd, *J* = 12.9, 11.6, 2.2 Hz, 1H), 4.15 (ddd, *J* = 11.7, 4.9, 1.9 Hz, 1H), 3.80 (s, 3H), 3.75 (s, 3H), 3.13 – 2.98 (m, 3H), 2.36 – 2.25 (m, 2H), 1.92 – 1.69 (m, 3H), 1.20 – 1.13 (m, 1H), 0.98 – 0.91 (m, 1H), 0.89 – 0.86 (m, 1H), 0.64 (s, 9H), –0.31 (s, 3H), –0.36 (s, 3H).

^{13}C NMR (100 MHz, CD_3OD) δ 192.7, 153.5, 151.7, 145.6, 136.9, 136.0, 134.6, 134.5, 134.0, 133.4, 130.0, 129.9, 127.8, 127.6, 125.3, 116.0, 110.9, 66.5, 61.2, 55.5, 55.1, 43.3, 38.7, 38.2, 37.7, 32.4, 26.7, 26.6, 25.0, 17.7, –6.8, –6.9.

IR (KBr): ν (cm^{-1}) 2922, 2851, 1697, 1658, 1632, 1587, 1470, 1428, 1252, 1218, 1191, 1150, 1109, 1093, 1033, 1010, 962, 942, 922, 851, 837, 804, 777, 735, 706, 684, 653, 605.

HRMS (ESI):*m/z* calcd. for $C_{38}H_{49}F_3O_5Si_2$ $[M+H]^+$: 641.3119, found 641.3104.

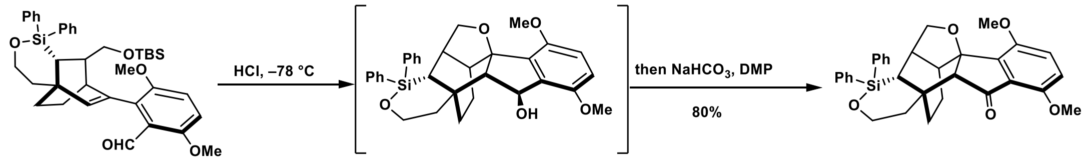

HCl (2M in EtOAc, 1.7 mL, 3.4 mmol) was added to a solution of (+)-**8** (210.0 mg, 0.33 mmol) in DCM (5 mL) at –78 ℃ under N_2 atmosphere. After stirring for 24 h at this temperature, the reaction was quenched by H_2O. The aqueous layer was extracted with DCM (three times). The combined extracts were dried over Na_2SO_4, filtered, and concentrated in vacuo to give crude alcohol which was used for the next step without further purification.

To a solution of crude alcohol, $NaHCO_3$ (277.0 mg, 10 mmol) in DCM (10 mL) at 0 °C was added Dess-Martin periodinane (279.2 mg, 0.66 mmol) and the mixture was warmed to room temperature. The reaction was completed in 30 min, quenched by saturated aq. $Na_2S_2O_3$ and extracted with DCM (three times). The combined organic layers were washed with brine, dried over anhydrous Na_2SO_4 and concentrated in *vacuo*. The resulting residue was purified by silica gel column chromatography (petroleum ether/ethyl acetate = 2:1 to 1:1) to afford (–)-**7** (138.6 mg, 80%) as a yellow solid.

Compound (–)-22

TLC (petroleum ether/ethyl acetate, 1:1 v/v): Rf = 0.35.

Optical rotation: $[\alpha]_D^{20}$ = –25 (c = 1.0, MeOH).

^{1}H NMR (500 MHz, $CDCl_3$) δ 7.79 – 7.76 (m, 2H), 7.60 – 7.57 (m, 2H), 7.44 – 7.40 (m, 3H), 7.40 – 7.37 (m, 2H), 7.36 – 7.34 (m, 1H), 6.73 (s, 2H), 5.74 (d, *J* = 8.8 Hz, 1H), 4.39 (ddd, *J* = 12.9, 11.5, 2.3 Hz, 1H), 4.17 (ddd, *J* = 11.5, 5.0, 1.8 Hz, 1H), 4.01 (dd, *J* = 7.1, 3.1 Hz, 1H), 3.81 (s, 3H), 3.77 (s, 3H), 3.53 (d, *J* = 7.1 Hz, 1H), 2.72 (q, *J* = 3.4 Hz, 1H), 2.62 (td, *J* = 4.0, 2.1 Hz, 1H), 2.32 (dt, *J* = 14.0, 2.1 Hz, 1H), 2.08 – 2.01 (m, 2H), 1.89 (td, *J* = 13.5, 5.0 Hz, 1H), 1.62 –

1.54 (m, 2H), 1.36 (t, J = 3.3 Hz, 1H), 1.31 (ddd, J = 11.2, 4.2, 2.3 Hz, 1H), 1.19 (dddd, J = 13.9, 11.2, 7.6, 2.1 Hz, 1H).

^{13}C NMR (125 MHz, $CDCl_3$) δ 151.6, 150.1, 136.4, 136.04, 136.03, 134.7, 134.6, 130.4, 130.1, 129.7, 128.1, 128.0, 111.7, 110.8, 93.8, 76.4, 74.4, 62.6, 62.0, 56.1, 55.5, 39.14, 39.11, 38.1, 36.7, 34.6, 22.7, 16.7.

IR (KBr): ν (cm^{-1}) 2923, 2853, 1495, 1463, 1428, 1282, 1260, 1186, 1112, 1075, 1093, 1040, 1019, 980, 943, 911, 863, 798, 759, 735, 702, 661.

HRMS (ESI):*m/z* calcd. for $C_{32}H_{35}O_5Si$ $[M+H]^+$: 527.2248, found 527.2253.

Compound (–)-7

TLC (petroleum ether/ethyl acetate, 1:1 v/v): Rf = 0.26.

Optical rotation: $[\alpha]_D^{20}$ = –13 (c = 1.0, MeOH).

^{1}H NMR (500 MHz, $CDCl_3$) δ 7.77 – 7.75 (m, 2H), 7.59 – 7.57 (m, 2H), 7.46 – 7.40 (m, 4H), 7.38– 7.35 (m, 2H), 7.05 (d, J = 8.8 Hz, 1H), 6.82 (d, J = 8.8 Hz, 1H), 4.33 (td, J = 12.4, 2.4 Hz, 1H), 4.20 (ddd, J = 11.7, 5.1, 1.8 Hz, 1H), 4.14 – 4.12 (m, 1H), 3.88 (s, 3H), 3.83 (s, 3H), 3.62 (d, J = 7.4 Hz, 1H), 2.76 (q, J = 3.4 Hz, 1H), 2.72 (dt, J = 14.5, 2.2 Hz, 1H), 2.59 (q, J = 3.3 Hz, 1H), 2.28 (d, J = 2.3 Hz, 1H), 1.99 – 1.92 (m, 1H), 1.67 – 1.59 (m, 1H), 1.47 (t, J = 3.0 Hz, 1H), 1.23 – 1.12 (m, 2H), 0.87 – 0.81 (m, 1H).

^{13}C NMR (125 MHz, $CDCl_3$) δ 201.1, 151.42, 151.41, 140.6, 135.7, 135.4, 134.60, 134.57, 130.4, 130.0, 128.2, 128.1, 127.9, 118.6, 111.8, 86.0, 77.2, 67.5, 61.9, 56.4, 56.1, 38.7, 38.5, 37.0, 36.6, 35.4, 21.2, 15.9.

IR (KBr): ν (cm^{-1}) 2925, 2866, 1707, 1590, 1495, 1464, 1428, 1286, 1266, 1238, 1195, 1113, 1092, 1078, 1061, 1039, 952, 939, 856, 811, 767, 741, 702, 620.

HRMS (ESI):*m/z* calcd. for $C_{32}H_{33}O_5Si$ $[M+H]^+$: 525.2097, found 525.2079.

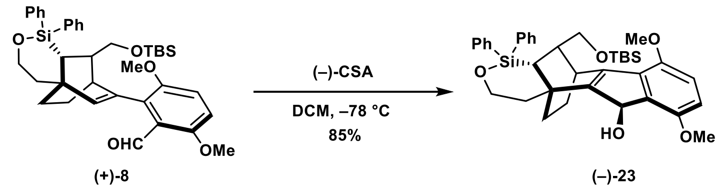

(–)-CSA (23.6 mg, 0.1 mmol) was added to a solution of (+)-**8** (64.5 mg, 0.1 mmol) in DCM (1.0 mL) at –78 ℃under N_2 atmosphere. After stirring for 12 h at this temperature, the reaction was quenched by H_2O. The aqueous layer was extracted with DCM (three times). The combined extracts were dried over Na_2SO_4, filtered, and concentrated in *vacuo*. The resulting residue was purified by silica gel column chromatography (petroleum ether/ethyl acetate = 8:1) to afford (–)-**23** (55.1 mg, 85%) as a yellow oil.

Compound (–)-23

TLC (petroleum ether/ethyl acetate, 5:1 v/v): Rf = 0.25.

Optical rotation: $[\alpha]_D^{20}$ = –55 (c = 1.0, MeOH).

^{1}H NMR (400 MHz, $CDCl_3$) δ 7.86 (dt, *J* = 5.0, 2.5 Hz, 2H), 7.61 – 7.55 (m, 2H), 7.50 (dd, *J* = 5.8, 1.7 Hz, 3H), 7.41 – 7.30 (m, 3H), 6.76 (d, *J* = 8.9 Hz, 1H), 6.63 (d, *J* = 8.8 Hz, 1H), 5.51 (s, 1H), 4.51 (td, *J* = 12.1, 2.8 Hz, 1H), 4.31 (ddd, *J* = 11.7, 5.0, 2.1 Hz, 1H), 3.89 (s, 3H), 3.78 (s, 3H), 3.73 (q, *J* = 2.9 Hz, 1H), 3.02 (dd, *J* = 10.0, 3.9 Hz, 1H), 2.82 (t, *J* = 9.5 Hz, 1H), 2.58 – 2.41 (m, 2H), 2.36 – 2.29 (m, 1H), 2.00 (td, *J* = 9.0, 8.3, 5.1 Hz, 1H), 1.42 (dt, *J* = 12.1, 4.0 Hz, 1H), 1.08 (tt, *J* = 13.6, 4.7 Hz, 1H), 0.95 – 0.89 (m, 1H), 0.77 (s, 9H), 0.70 – 0.64 (m, 1H), **–**0.18 (s, 3H), **–**0.21 (s, 3H).

^{13}C NMR (100 MHz, $CDCl_3$) δ 151.4, 150.2, 148.5, 142.4, 136.2, 134.9, 134.5, 133.2, 131.5, 130.0, 130.0, 129.8, 127.9, 127.8, 113.2, 108.7, 73.6, 66.7, 61.5, 56.3, 55.7, 43.8, 39.1, 35.3, 33.1, 32.9, 27.7, 26.4, 25.8, 18.2, –5.6, –5.9.

IR (KBr): ν (cm^{-1}) 3480, 2935, 2855, 1494, 1463, 1428, 1257, 1185, 1112, 1057, 1033, 867, 837, 777, 736, 721, 702, 661, 620.

HRMS (ESI):*m/z* calcd. for $C_{38}H_{48}O_5NaSi_2$ $[M+Na]^+$: 663.2932, found 663.2932.

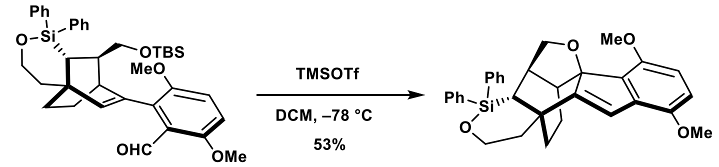

TMSOTf (18 μL, 0.1 mmol) was added to a solution of (+)-**8** (64.2 mg, 0.1 mmol) in DCM (5.0 mL) at –78 ℃under N_2 atmosphere. After stirring for 12 h at this temperature, the reaction was quenched by H_2O. The aqueous layer was extracted with DCM (three times). The combined extracts were dried over Na_2SO_4, filtered, and concentrated in *vacuo*. The resulting residue was purified by silica gel column chromatography (petroleum ether/ethyl acetate = 5:1) to afford (–)-**25** (27.1 mg, 53%) as a yellow soild.

Compound (–)-25

TLC (petroleum ether/ethyl acetate, 5:1 v/v): Rf = 0.30.

Optical rotation: $[\alpha]_D^{20}$ = –23 (c = 1.0, MeOH).

^{1}H NMR (400 MHz, $CDCl_3$) δ 7.82 – 7.76 (m, 2H), 7.58 (dt, *J* = 6.5, 1.7 Hz, 2H), 7.48 – 7.42 (m, 3H), 7.42 – 7.33 (m, 3H), 6.73 (d, *J* = 8.9 Hz, 1H), 6.59 (d, *J* = 8.9 Hz, 1H), 6.28 (s, 1H), 4.38 (td, *J* = 13.0, 12.3, 2.4 Hz, 1H), 4.27 (ddd, *J* = 11.7, 5.2, 1.9 Hz, 1H), 4.06 (dd, *J* = 6.8, 2.5 Hz, 1H), 3.80 (s, 3H), 3.78 (s, 3H), 3.54 (d, *J* = 6.8 Hz, 1H), 3.05 – 3.00 (m, 1H), 2.85 (q, *J* = 3.6 Hz, 1H), 2.26 (td, *J* = 13.6, 13.0, 5.2 Hz, 1H), 2.11 – 2.03 (m, 1H), 1.97 (dt, *J* = 14.2, 2.2 Hz, 1H), 1.87 (t, *J* = 3.2 Hz, 1H), 1.61 – 1.54 (m, 1H), 1.17 – 1.09 (m, 2H).

^{13}C NMR (100 MHz, $CDCl_3$) δ 159.9, 151.3, 147.6, 135.5, 135.4, 134.6, 134.5, 133.9, 131.0, 130.3, 129.9, 128.1, 128.0, 116.6, 113.4, 110.0, 92.3, 76.1, 61.6, 56.3, 56.1, 40.5, 38.1, 37.2, 35.6, 34.9, 32.4, 16.6.

IR (KBr): ν (cm^{-1}) 2921, 2851, 1659, 1632, 1494, 1462, 1428, 1289, 1271, 1259, 1211, 1084, 1112, 1081, 1038, 1010, 979, 965, 942, 924, 897, 865, 844, 807, 767, 737, 708, 681.

HRMS (ESI):*m/z* calcd. for $C_{32}H_{33}O_4Si$ $[M+H]^+$: 509.2142, found 509.2147.

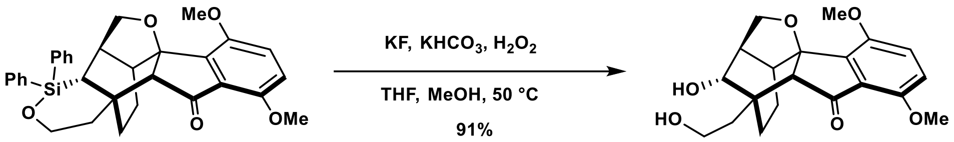

(–)-7 (–)-26

To a solution of (–)-**7** (131.4 mg, 0.25 mmol), KF (72.5 mg, 1.25 mmol), $KHCO_3$ (40.1 mg, 0.4 mmol) in MeOH/THF (1:1, 5.0 mL) was added H_2O_2 (30% wt with H_2O, 0.64 mL, 5.0 mmol) at room temperature. The mixture was warmed to 50 ℃, and stirred for 12 h. Then the reaction mixture was cooled to 0 ℃, quenched by saturated aq. $Na_2S_2O_3$ and extracted with EtOAc (three times). The combined organic layers were washed with brine, dried over anhydrous Na_2SO_4, filtered, and concentrated in *vacuo*. The resulting residue was purified by silica gel column chromatography (pure ethyl acetate) to afford (–)-**26** (83.3 mg, 91%) as a colorless oil.

Compound (–)-26

TLC (pure ethyl acetate): Rf = 0.4.

Optical rotation: $[\alpha]_D^{20}$ = –24 (c = 1.0, MeOH).

^{1}H NMR (500 MHz, $CDCl_3$) δ 7.06 (d, *J* = 8.8 Hz, 1H), 6.83 (d, *J* = 8.8 Hz, 1H), 4.15 (dd, *J* = 7.9, 4.4 Hz, 1H), 3.88 (s, 3H), 3.83 (s, 3H), 3.81 (dd, *J* = 6.1, 3.2 Hz, 1H), 3.77 – 3.72 (m, 2H), 3.61 (s, 1H), 2.65 – 2.63 (m, 1H), 2.41 (ddd, *J* = 15.6, 6.0, 2.4 Hz, 1H), 2.33 (t, *J* = 4.2 Hz, 1H), 2.19 (d, *J* = 1.8 Hz, 1H), 1.96 (ddd, *J* = 15.6, 9.3, 3.2 Hz, 1H), 1.79 – 1.69 (m, 2H), 1.05 – 0.96 (m, 2H).

^{13}C NMR (125 MHz, $CDCl_3$) δ 201.7, 151.4, 151.3, 140.0, 128.0, 118.8, 112.0, 86.1, 78.2, 73.6, 59.5, 58.5, 56.4, 56.0, 46.0, 39.3, 37.5, 36.4, 17.9, 15.8.

IR (KBr): ν (cm^{-1}) 3375, 2962, 2921, 2851, 1704, 1497, 1465, 1260, 1099, 1032, 860, 799, 706, 625, 606.

HRMS (ESI):*m/z* calcd. for $C_{20}H_{25}O_6$ $[M+H]^+$: 361.1651, found 361.1638.

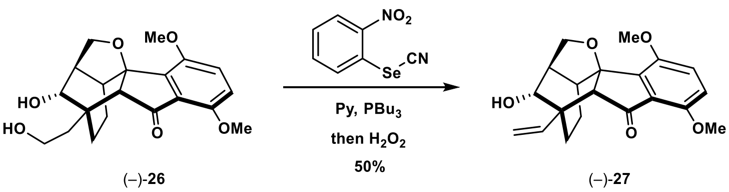

(–)-26 (–)-27

To a solution of (–)-**26** (73.5 mg, 0.2 mmol), 2-nitrophenylselenocyanate (55.1 mg, 0.24 mmol) and pyridine (20 μL, 0.24 mmol) in dry THF (5.0 mL) PBu_3 (60 μL, 0.24 mmol) was added dropwise at room temperature. Upon consumption of the starting material as indicated by TLC, H_2O_2 (30% wt with H_2O, 0.6 mL, 4.8 mmol) was added into this mixture and stirred for 12 h. Then the reaction mixture was cooled to 0 ℃, quenched by saturated aq. $Na_2S_2O_3$ and extracted with EtOAc (three times). The combined organic layers were washed with brine, dried over anhydrous Na_2SO_4, filtered, and concentrated in *vacuo*. The resulting residue was purified by silica gel column chromatography (petroleum ether/ethyl acetate = 1:1) to afford (–)-**27** (34.0 mg, 50%) as a colorless solid.

Compound (–)-27

TLC (petroleum ether/ethyl acetate, 1:1 v/v): Rf = 0.15.

Optical rotation: $[\alpha]_D^{20}$ = –40 (c = 1.0, $CHCl_3$).

^{1}H NMR (500 MHz, $CDCl_3$) δ 7.07 (d, *J* = 8.9 Hz, 1H), 6.85 (d, *J* = 8.8 Hz, 1H), 6.01 (dd, *J* = 17.6, 10.9 Hz, 1H), 5.35 (dd, *J* = 10.9, 1.0 Hz, 1H), 5.13 (dd, *J* = 17.6, 1.1 Hz, 1H), 4.22 (dd, *J* = 8.1, 4.5 Hz, 1H), 3.88 (s, 3H), 3.85 (s, 3H), 3.79 – 3.76 (d, *J* = 7.6 Hz, 1H), 3.55 (s, 1H), 2.70 (q, *J* = 3.2 Hz, 1H), 2.42 (t, *J* = 4.2 Hz, 1H), 2.33 (d, *J* = 1.8 Hz, 1H), 1.92 (d, *J* = 1.6 Hz, 1H), 1.83 – 1.74 (m, 2H), 1.23 – 1.17 (m, 1H), 1.10 – 1.02 (m, 1H).

^{13}C NMR (125 MHz, $CDCl_3$) δ 199.6, 151.52, 151.45, 142.4, 139.6, 128.1, 118.8, 116.0, 112.4, 85.8, 73.6, 61.9, 56.5, 56.5, 56.1, 44.8, 42.6, 37.8, 15.48, 15.47.

IR (KBr): ν (cm^{-1}) 3402, 2963, 2921, 2851, 1705, 1591, 1496, 1463, 1418, 1264, 1100, 1055, 1032, 913, 868, 806, 708, 662.

HRMS (ESI):*m/z* calcd. for $C_{20}H_{23}O_5$ $[M+H]^+$: 343.1545, found 343.1534.

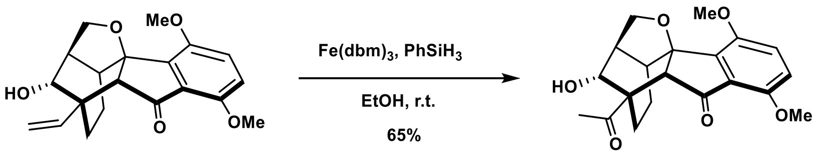

(–)-**27** (–)-**28**

To a solution of (–)-**27** (34.0 mg, 0.1 mmol) in EtOH (3.0 mL) was added $FeCl_3$ (1.7 mg, 0.01 mmol) followed by the addition of dibenzoylmethane (2.3mg, 0.01 mmol) while the reaction mixture was stirred open to air. After 5 min, $PhSiH_3$ (120 μL, 1.0 mmol) was added in one portion, and the solution was stirred at room temperature for 2 h. The reaction mixture was then quenched by water, extracted with EtOAc (three times). The combined organic layers were washed with brine, dried over anhydrous Na_2SO_4, filtered, and concentrated *in vacuo*. The crude product was purified by silica gel column chromatography (petroleum ether/ethyl acetate = 1:3) to afford (–)-**28** (23.1 mg, 65%) as a colorless solid.

Compound (–)-28

TLC (pure ethyl acetate): Rf = 0.25.

Optical rotation: $[\alpha]_D^{20}$ = –41 (c = 1.0, $CHCl_3$).

^{1}H NMR (500 MHz, $CDCl_3$) δ 7.10 (d, *J* = 8.2 Hz, 1H), 6.68 (d, *J* = 8.8 Hz, 1H), 4.21 (dd, *J* = 8.3, 4.6 Hz, 1H), 3.97 – 3.95 (m, 1H), 3.90 (s, 3H), 3.85 (s, 3H), 3.79 (d, J = 8.2 Hz, 1H), 2.92 (d, *J* = 2.3 Hz, 1H), 2.71 (q, *J* = 3.4 Hz, 1H), 2.42 - 2.36 (m, 1H), 2.38 (s, 3H), 2.19 – 2.12 (m, 1H), 2.01 (q, *J* = 6.8 Hz, 1H), 1.83 (ddt, *J* = 14.4, 12.1, 3.9 Hz, 1H), 1.45 – 1.40 (m, 1H), 1.07 (ddd, *J* = 14.3, 5.1, 2.6 Hz, 1H).

^{13}C NMR (125 MHz, $CDCl_3$) δ 213.7, 199.4, 151.52, 151.46, 139.5, 127.5, 119.2, 112.4, 85.4, 77.8, 73.1, 61.6, 56.5, 56.1, 51.3, 47.4, 37.7, 28.8, 15.4, 14.9.

IR (KBr): ν (cm^{-1}) 3355, 2955, 2923, 2853, 1709, 1593, 1497, 1462, 1288, 1271, 1261, 1097, 1053, 1032, 801, 708.

HRMS (ESI):*m/z* calcd. for $C_{20}H_{23}O_6$ $[M+H]^+$: 359.1495, found 359.1551.

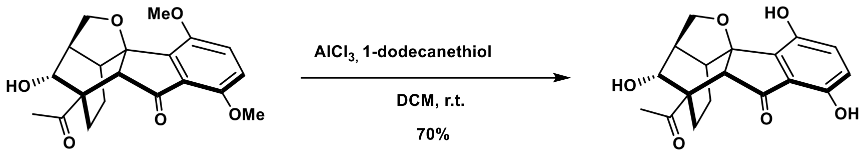

(–)-**28** (–)-**lucidumone, 1**

To a Schlenk tube were added $AlCl_3$ (330.2 mg, 2.5 mmol) under N_2 atmosphere followed by the addition of (–)-**28** (17.5 mg in 2.0 mL DCM, 0.05 mmol) at 0 ℃, then 1-dodecanethiol (0.6 mL, 2.5 mmol) was added to this mixture, and the reaction mixture was allowed to stir at 25 °C for 5 h. Upon completion, the solution was quenched by water (5.0 mL), extracted with EtOAc (three times), and the combined organic layers were washed with brine, dried over anhydrous Na_2SO_4, filtered, and concentrated *in vacuo*. The crude product was purified by silica gel column chromatography (petroleum ether/ethyl acetate = 1:1) to afford (**–**)-**lucidumone** (8.1 mg, 70%) as a brown solid.

(–)-Lucidumone [(–)-(1)]

TLC (petroleum ether/ethyl acetate, 1:1 v/v): Rf = 0.10.

Optical rotation: $[\alpha]_D^{20}$ = –62 (c = 1.0, MeOH).

^{1}H NMR (500 MHz, CD_3OD) δ 7.02 (d, *J* = 8.8 Hz, 1H), 6.76 (d, *J* = 8.7 Hz, 1H), 4.26 (dd, *J* = 8.3, 4.9 Hz, 1H), 3.92 – 3.89 (m, 1H), 3.78 (d, *J* = 8.4 Hz, 1H), 2.89 (d, *J* = 2.3 Hz, 1H), 2.70 (q-like, (3.3) 1H), 2.36 (t-like, (4.6) 1H), 2.29 (s, 3H), 2.21 – 2.15 (m, 1H), 1.92 – 1.83 (m, 1H), 1.38 – 1.32 (m, 1H), 1.07 (dddd, *J* = 14.7, 11.9, 4.9, 2.7 Hz, 1H).

^{13}C NMR (125 MHz, CD_3OD) δ 214.8, 204.3, 150.8, 149.5, 135.4, 126.4, 125.4, 118.6, 87.4, 79.5, 74.3, 62.4, 52.7, 48.7, 39.1, 28.5, 16.2, 15.9.

IR (KBr): ν (cm^{-1}) 3355, 3186, 2919, 2849, 1697, 1659, 1632, 1495, 1470, 1423, 1296, 1262, 1190,1051.

HRMS (ESI):*m/z* calcd. for $C_{18}H_{19}O_6$ $[M+H]^+$: 331.1182, found 331.1177.

3.2 NMR spectra and HPLC spectra

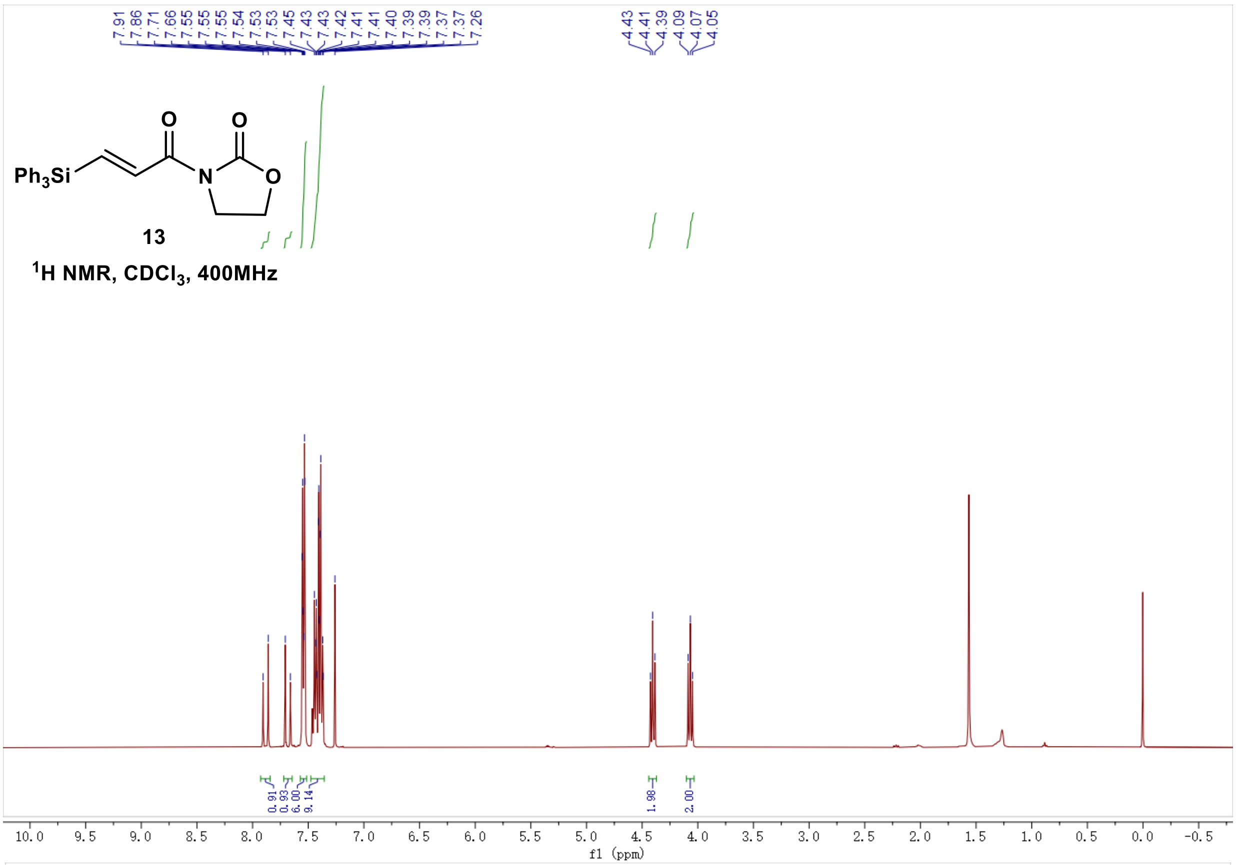

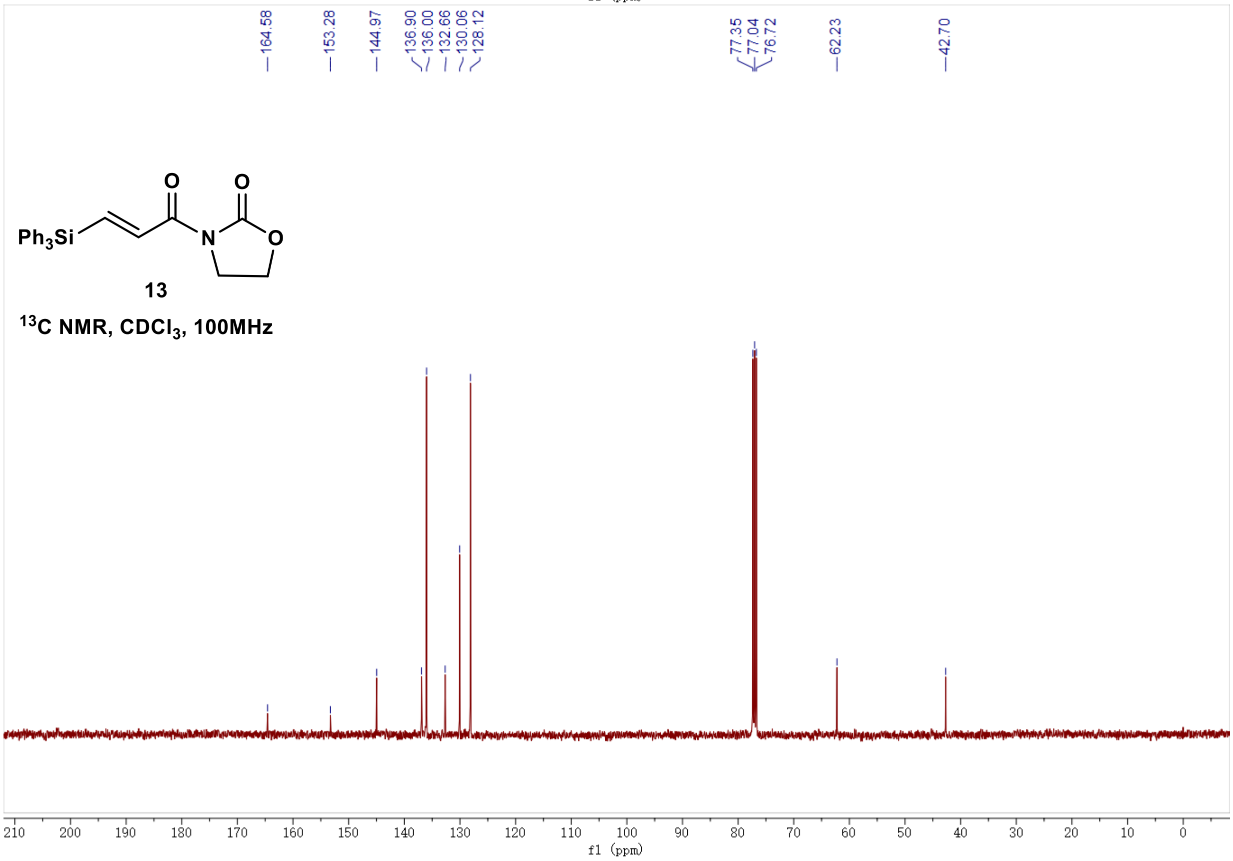

Supplementary Figure 3. NMR spectra of compound **13**

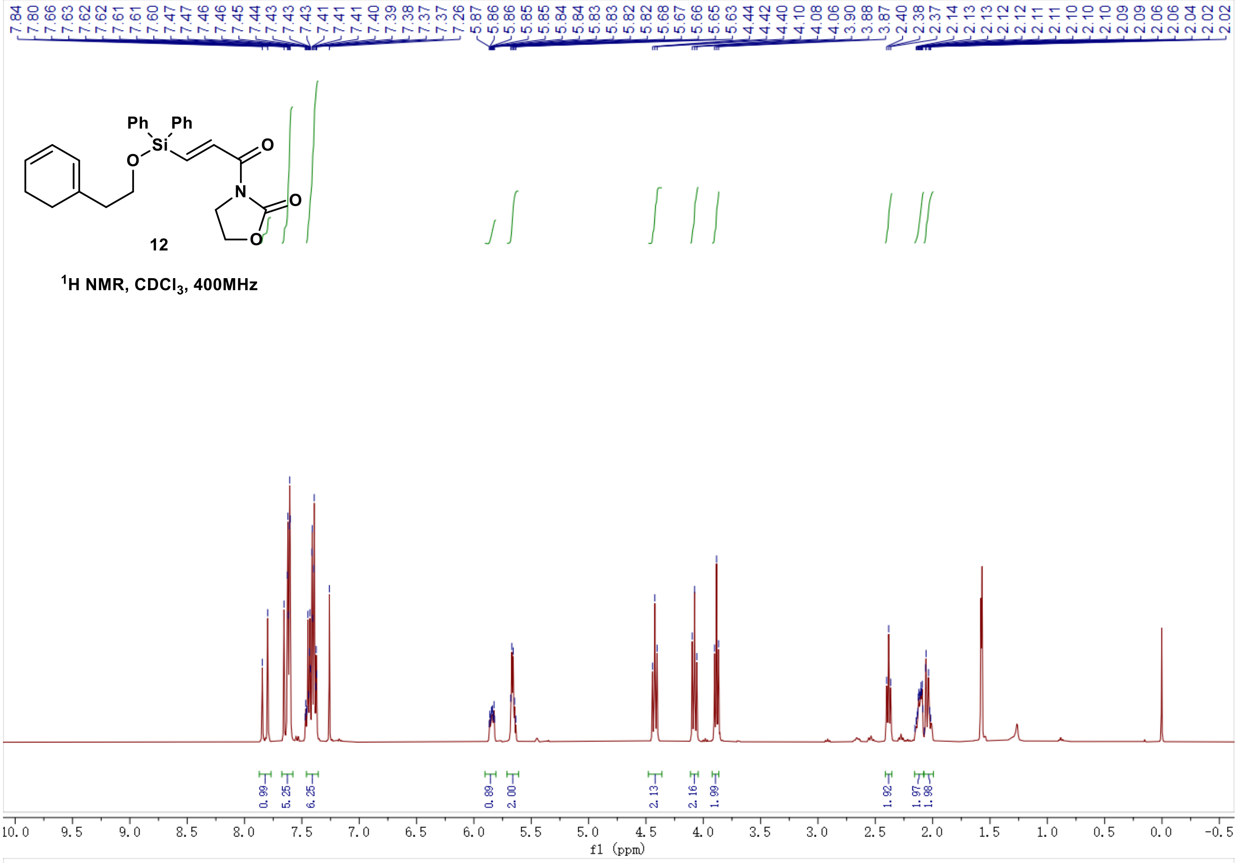

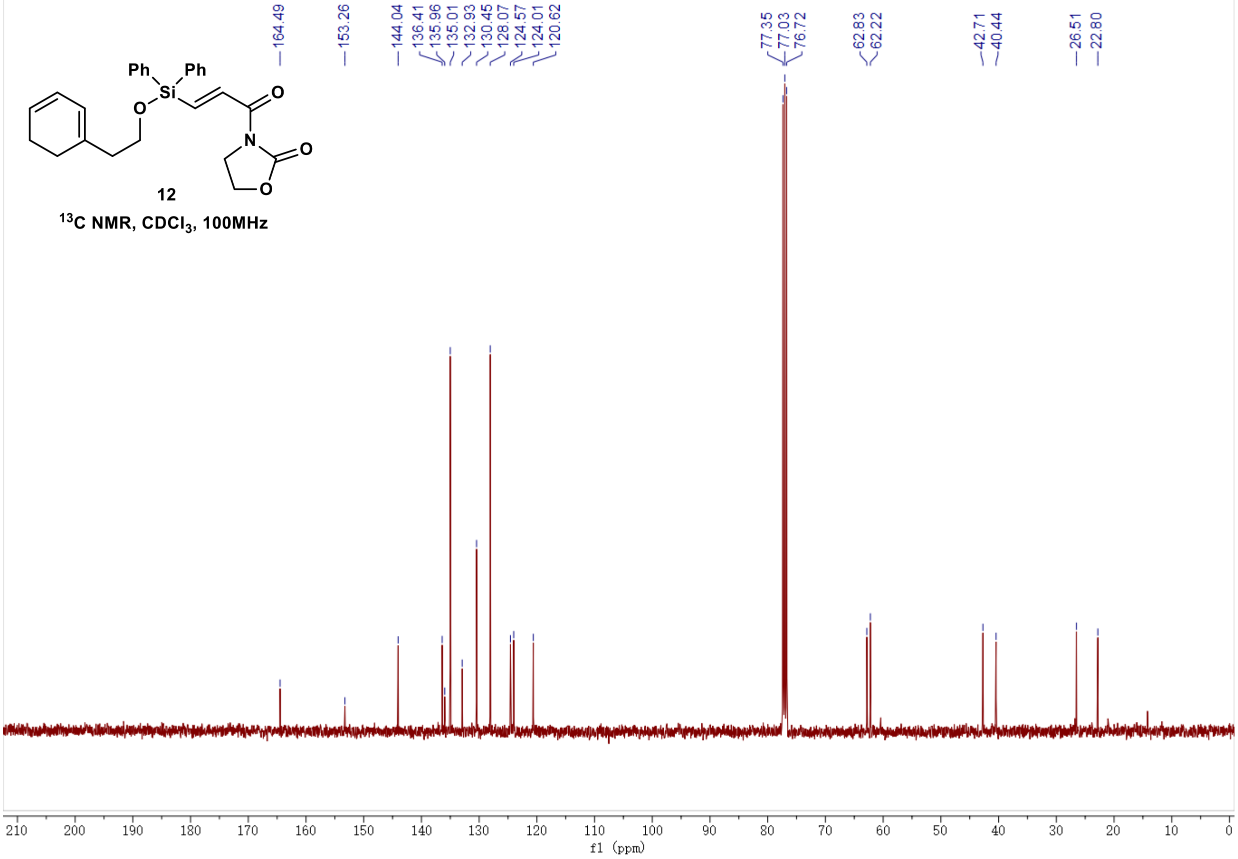

Supplementary Figure 4. NMR spectra of compound **12**

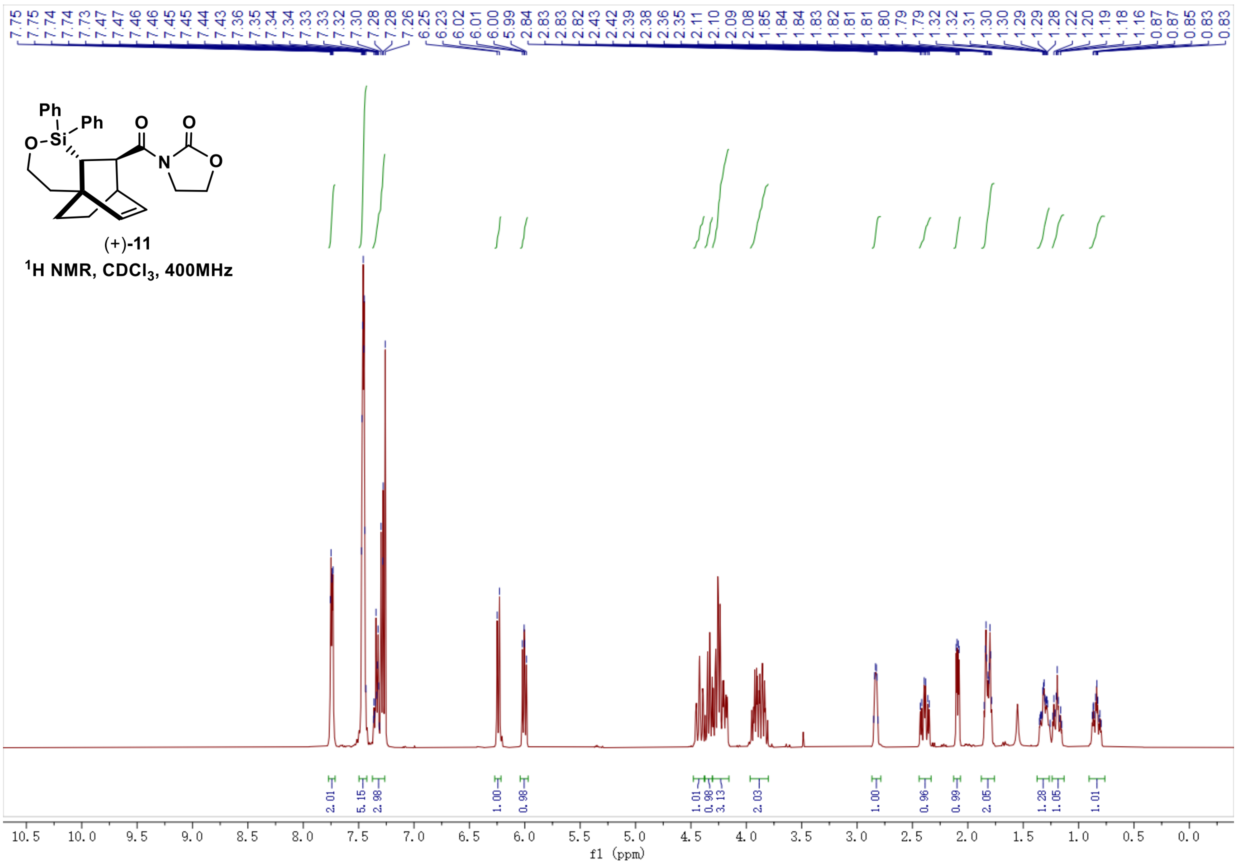

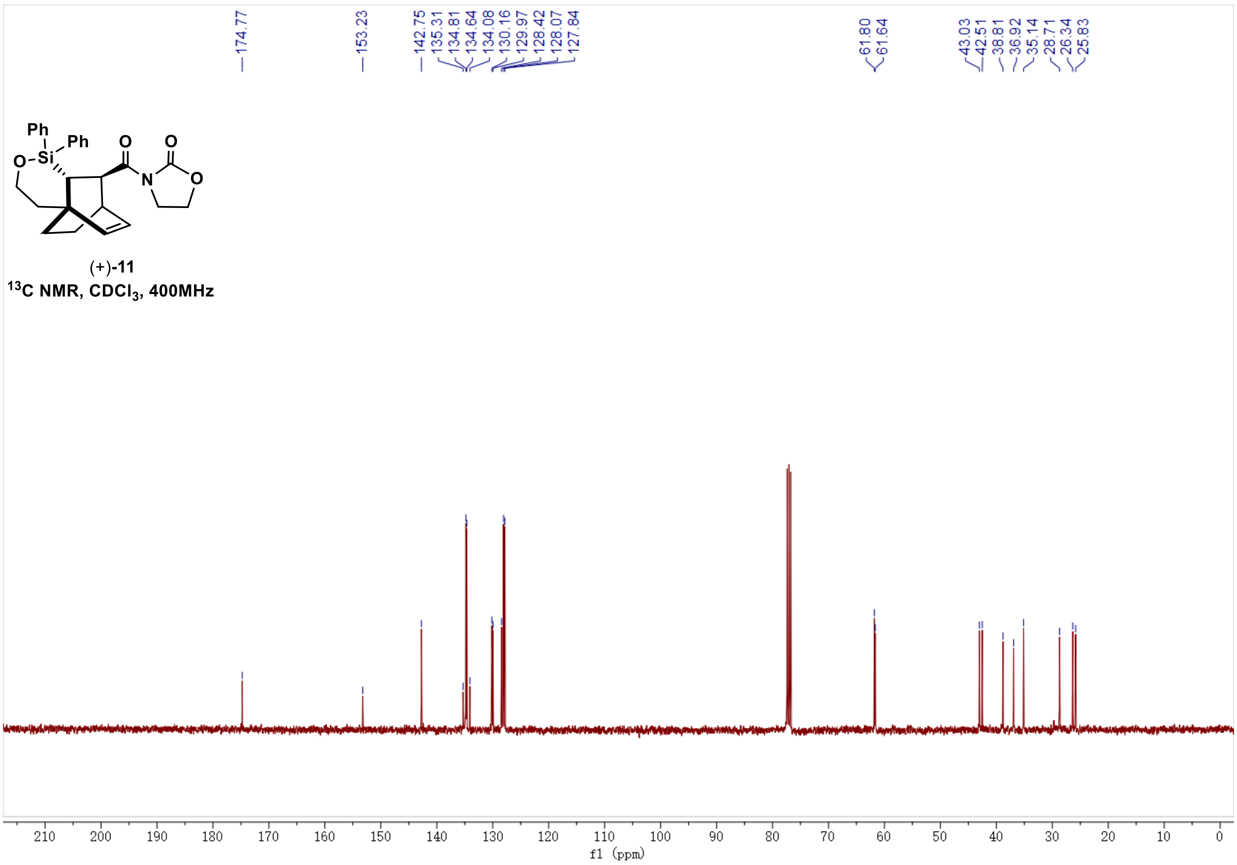

Supplementary Figure 5. NMR spectra of compound **(+)-11**

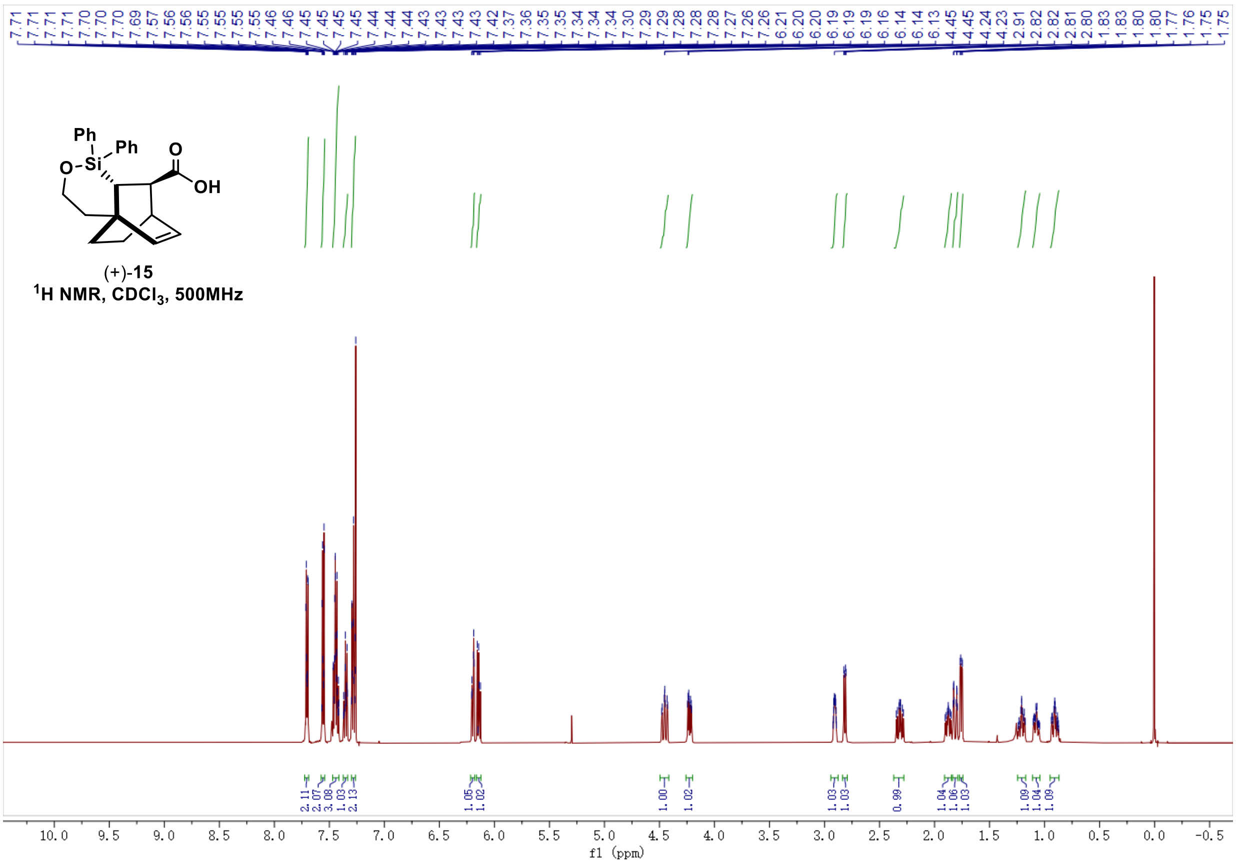

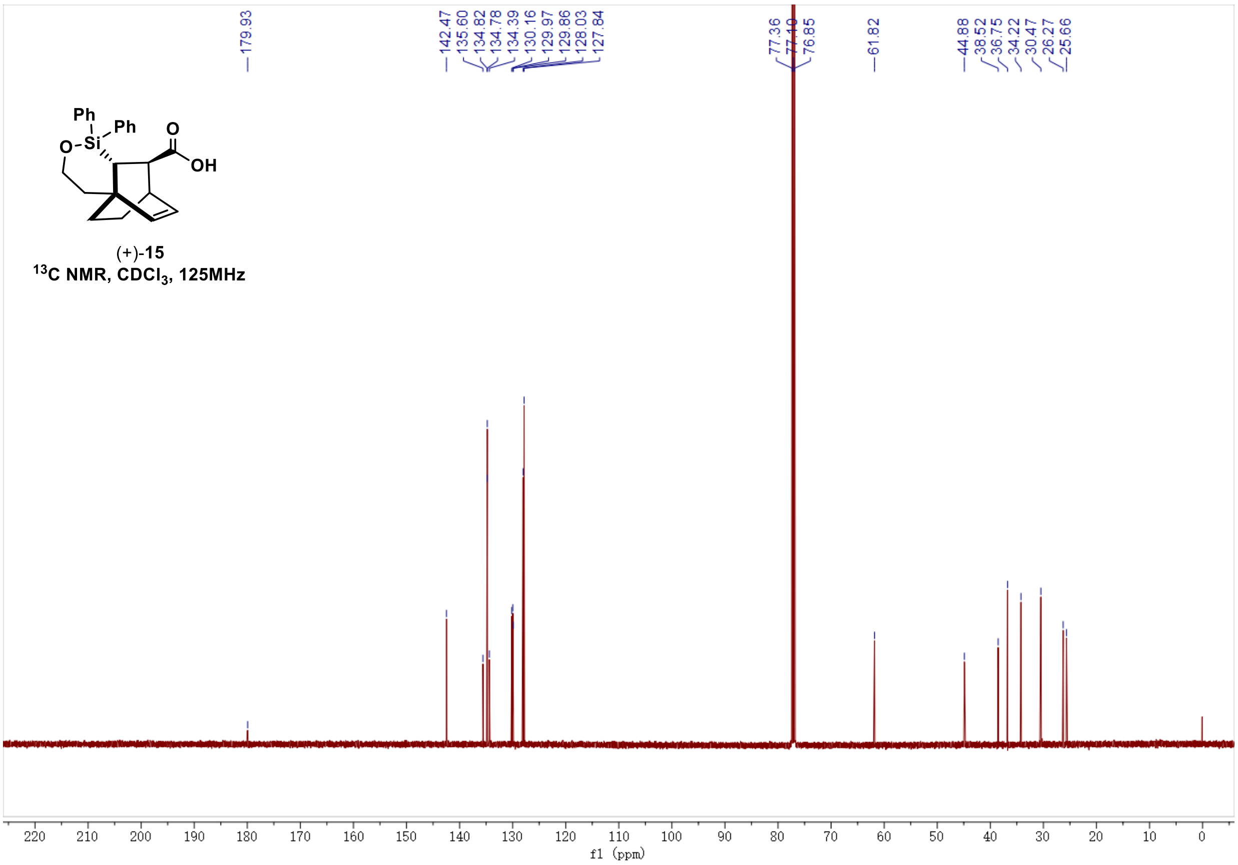

Supplementary Figure 6. NMR spectra of compound **(+)-15**

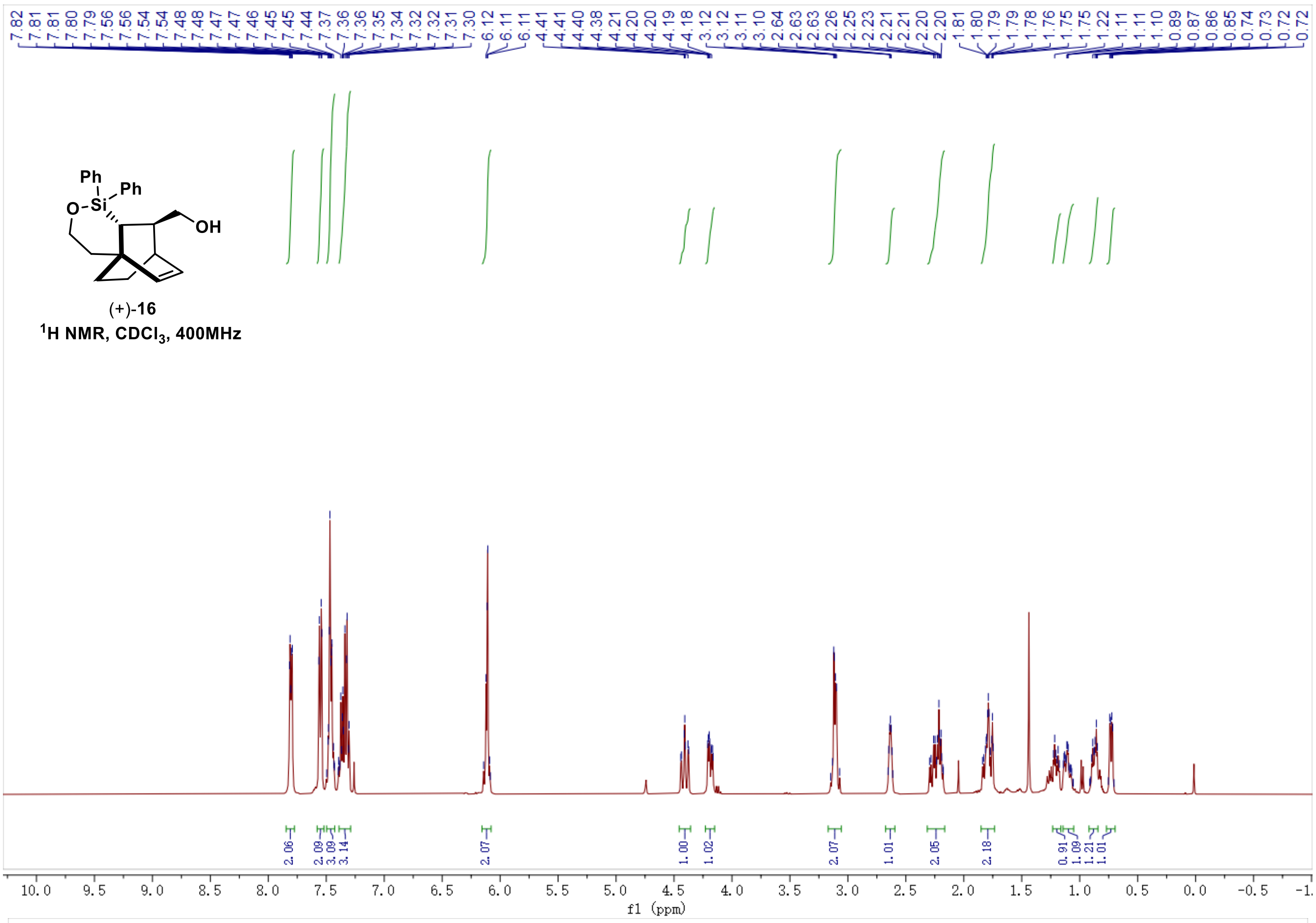

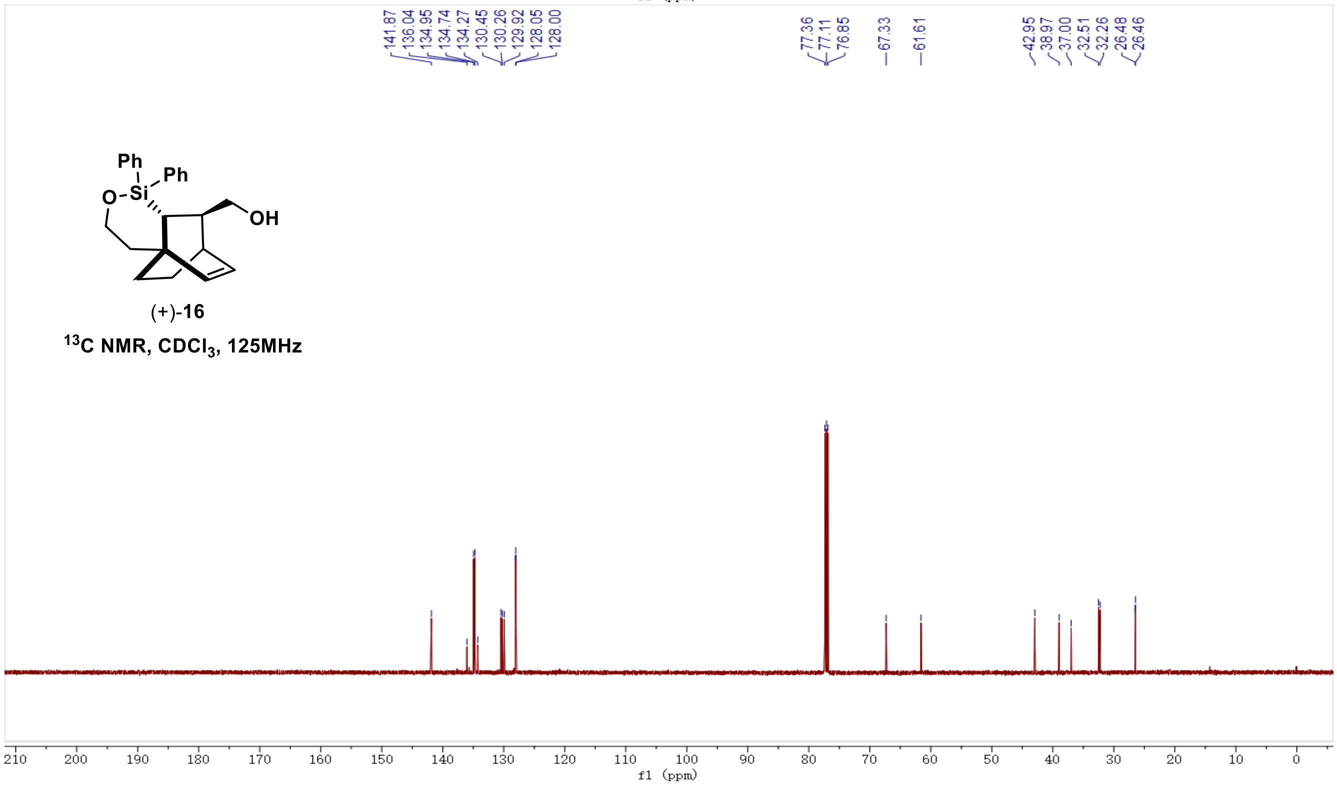

Supplementary Figure 7. NMR spectra of compound **(+)-16**

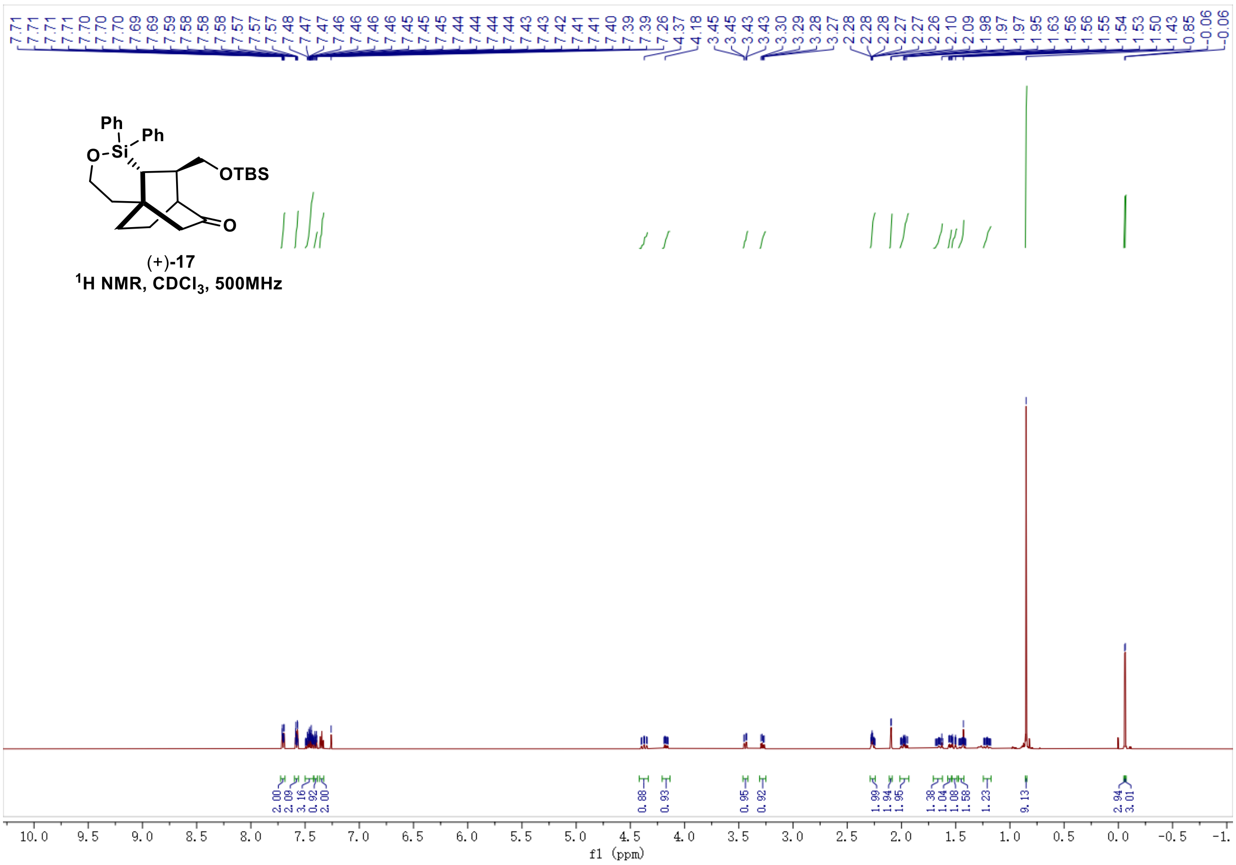

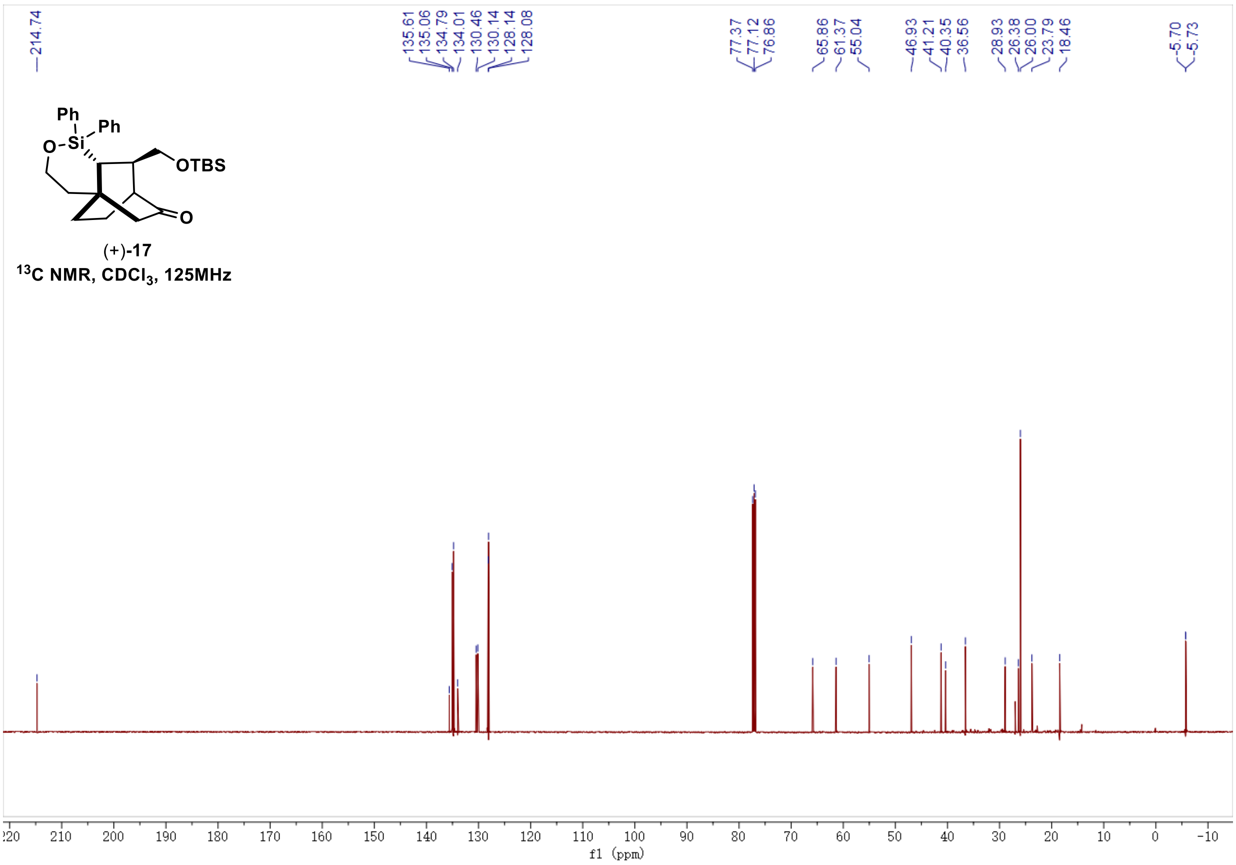

Supplementary Figure 8. NMR spectra of compound **(+)-17**

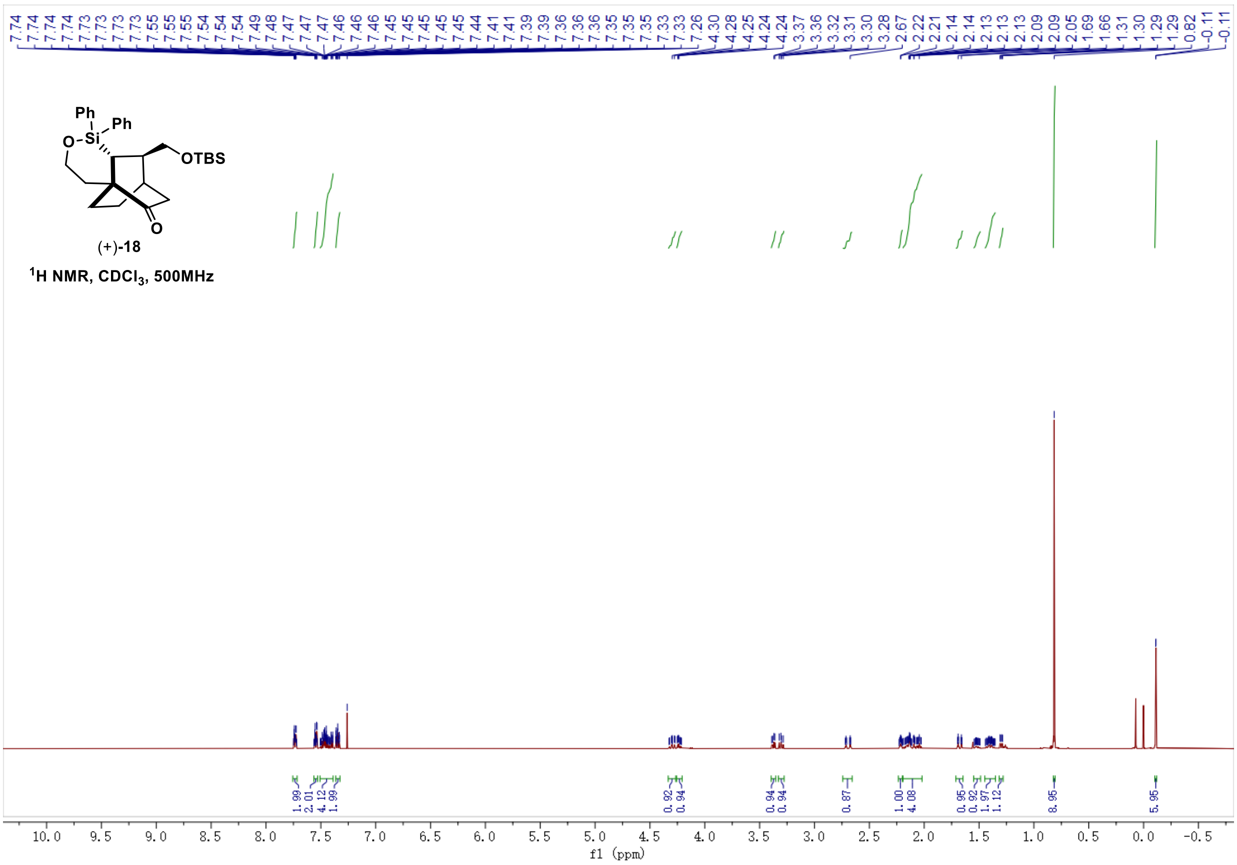

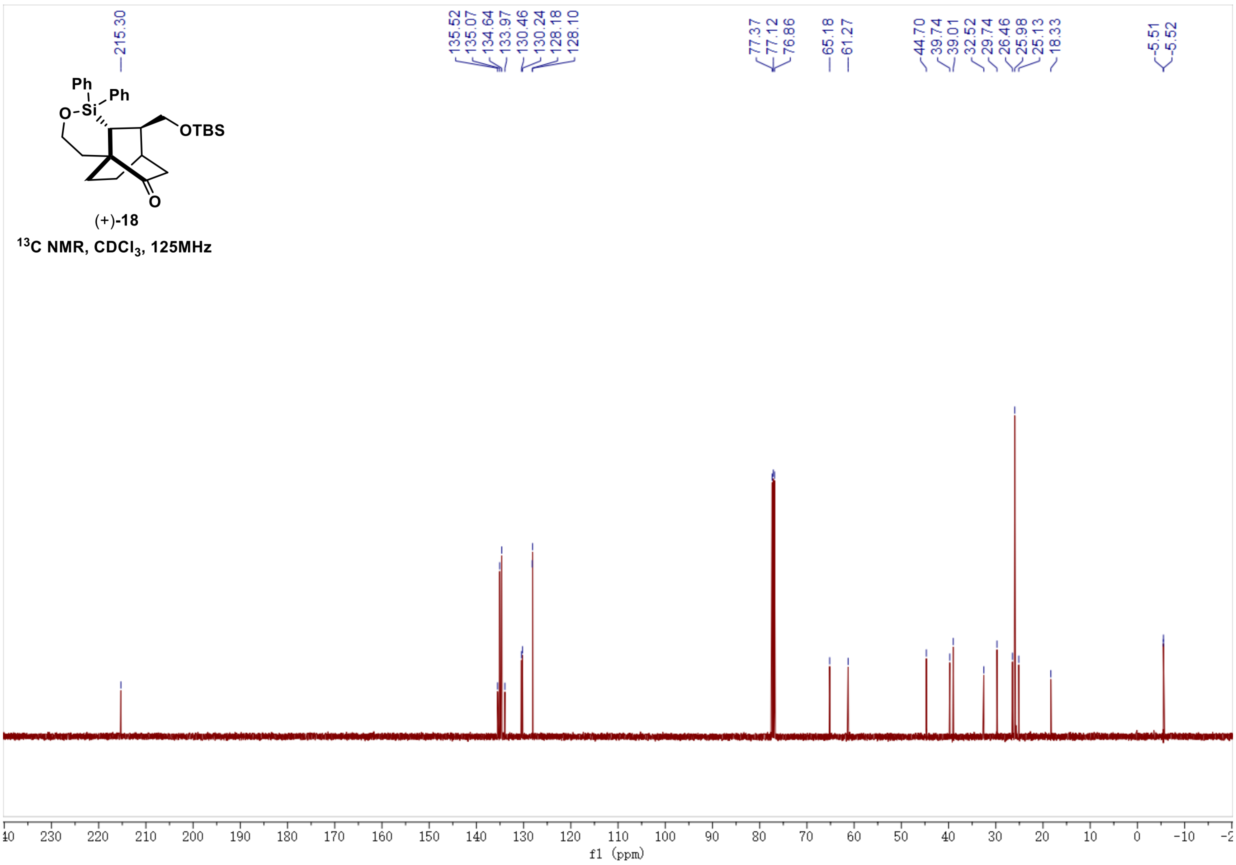

Supplementary Figure 9. NMR spectra of compound **(+)-18**

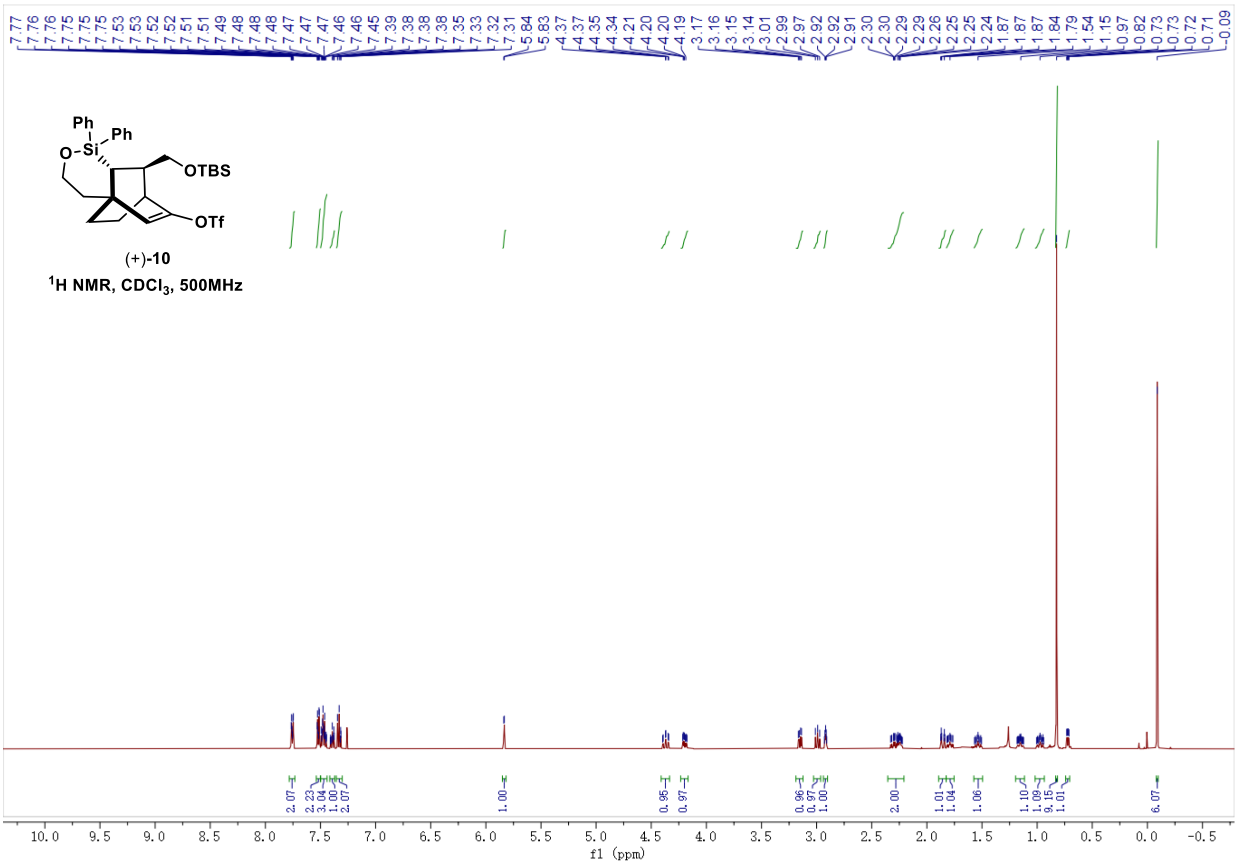

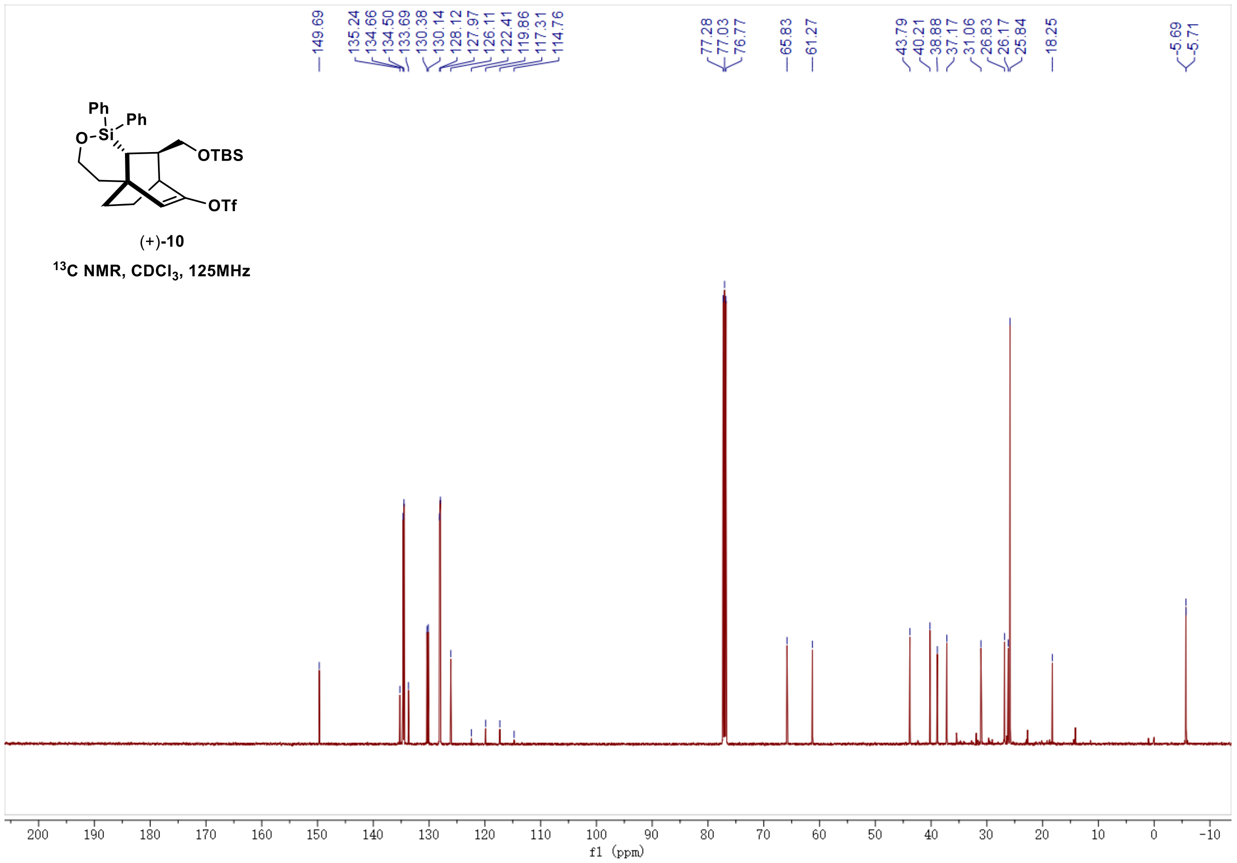

Supplementary Figure 10. NMR spectra of compound **(+)-10**

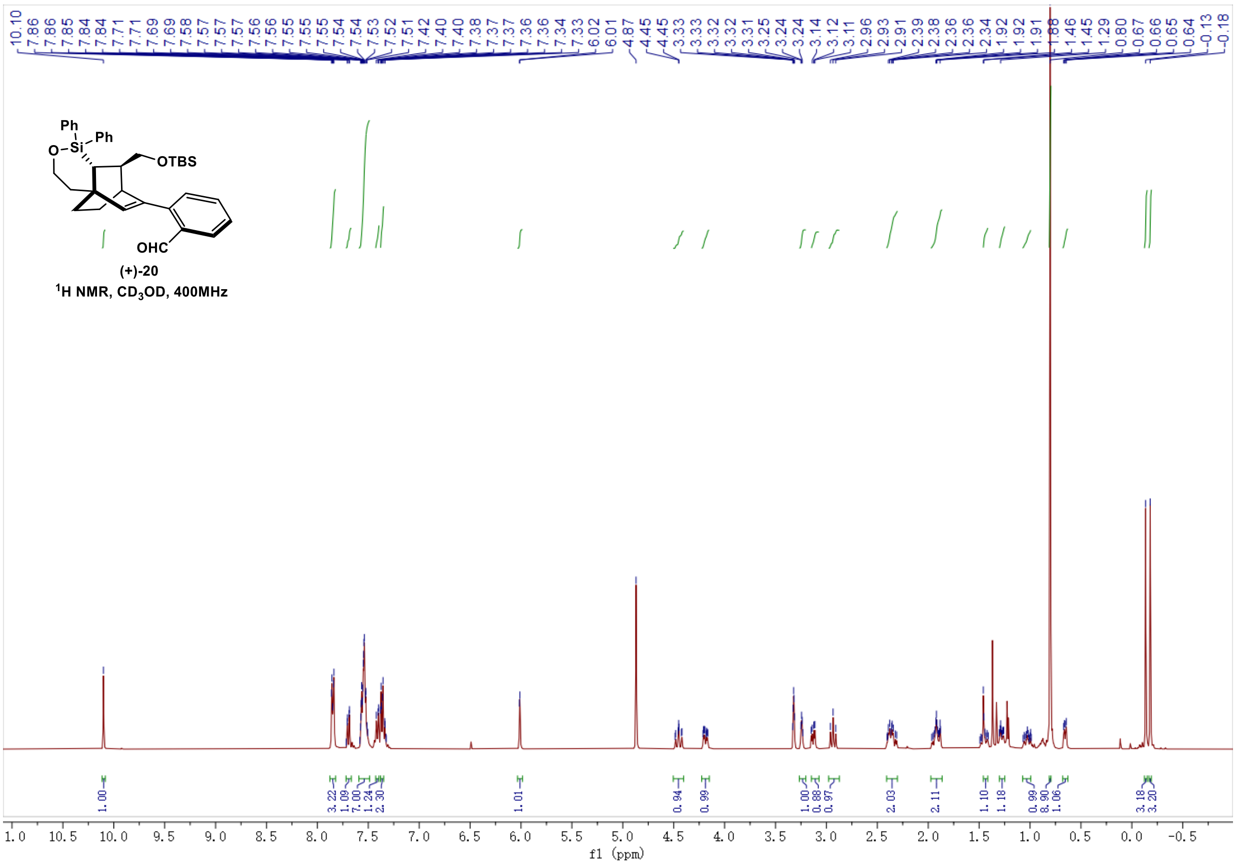

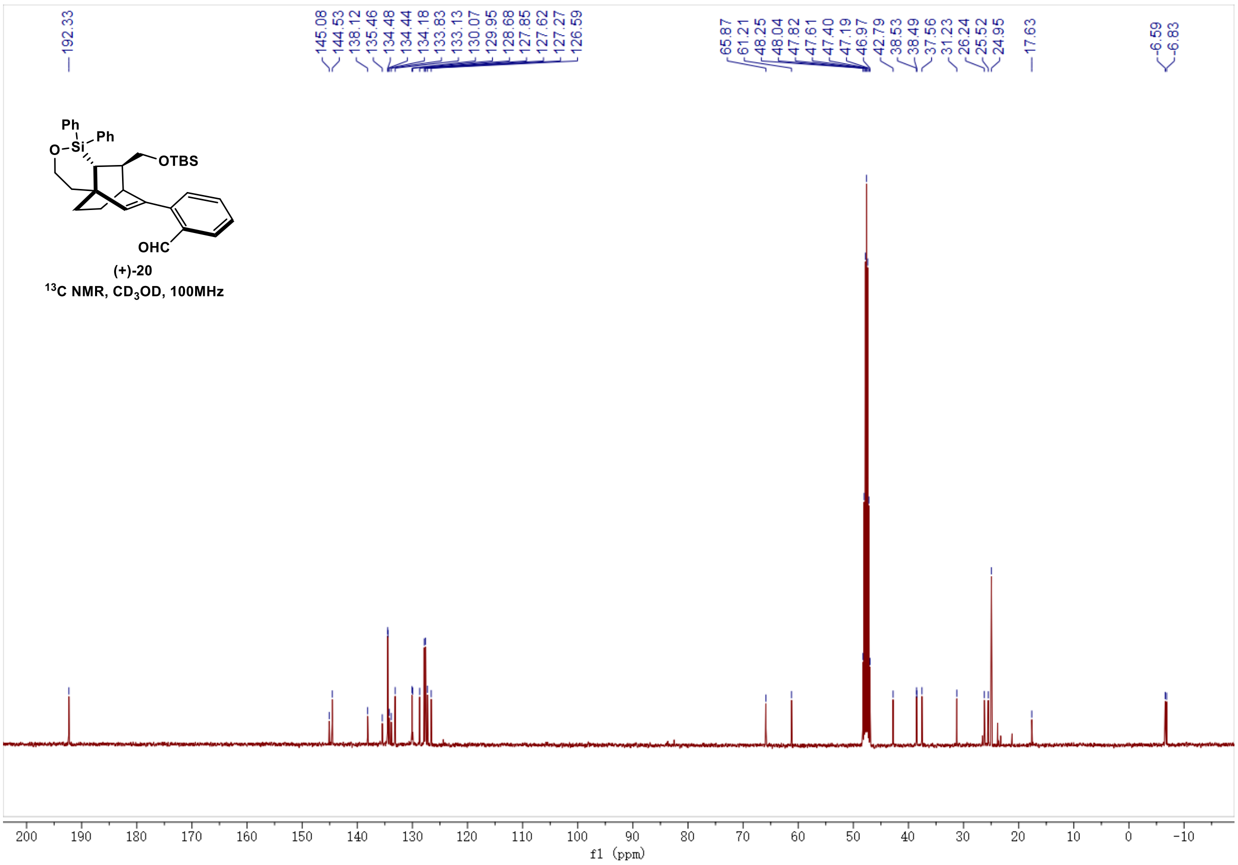

Supplementary Figure 11. NMR spectra of compound **(+)-20**

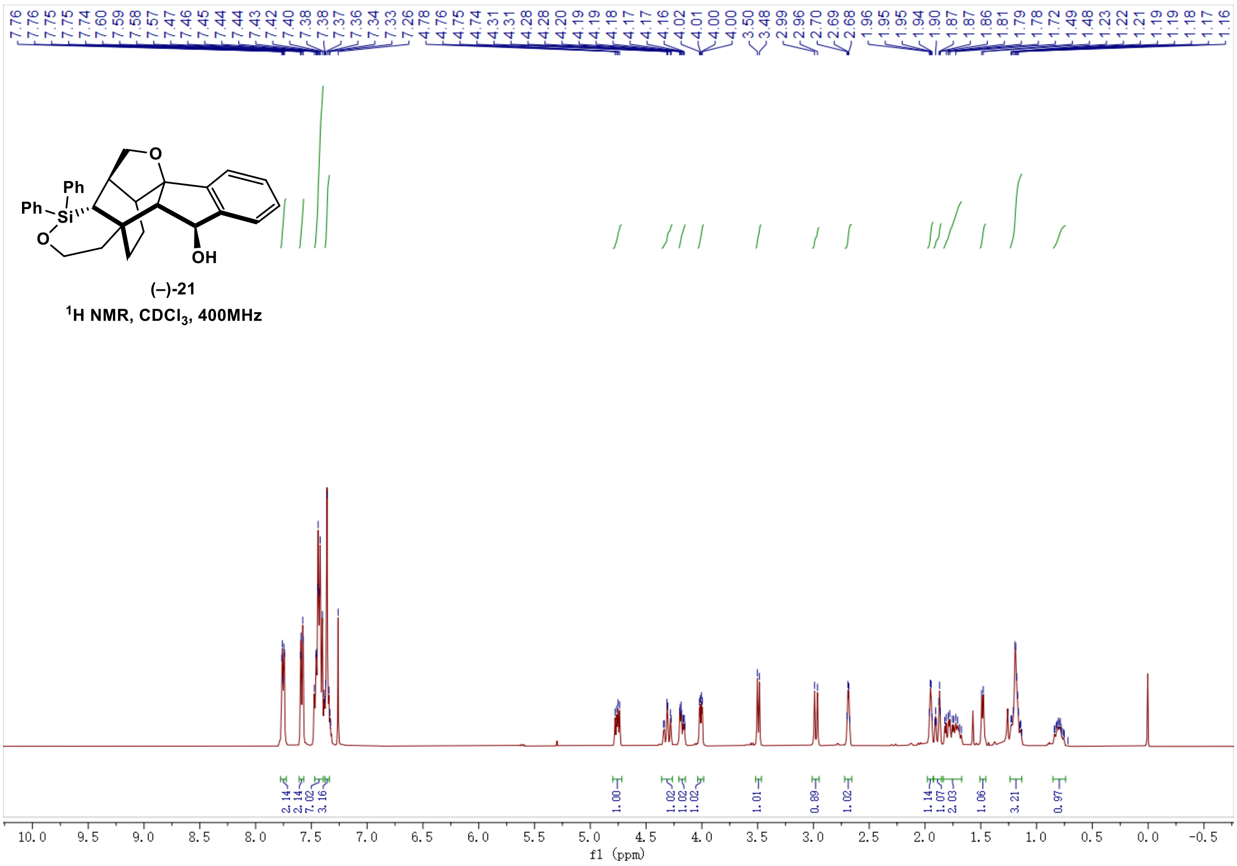

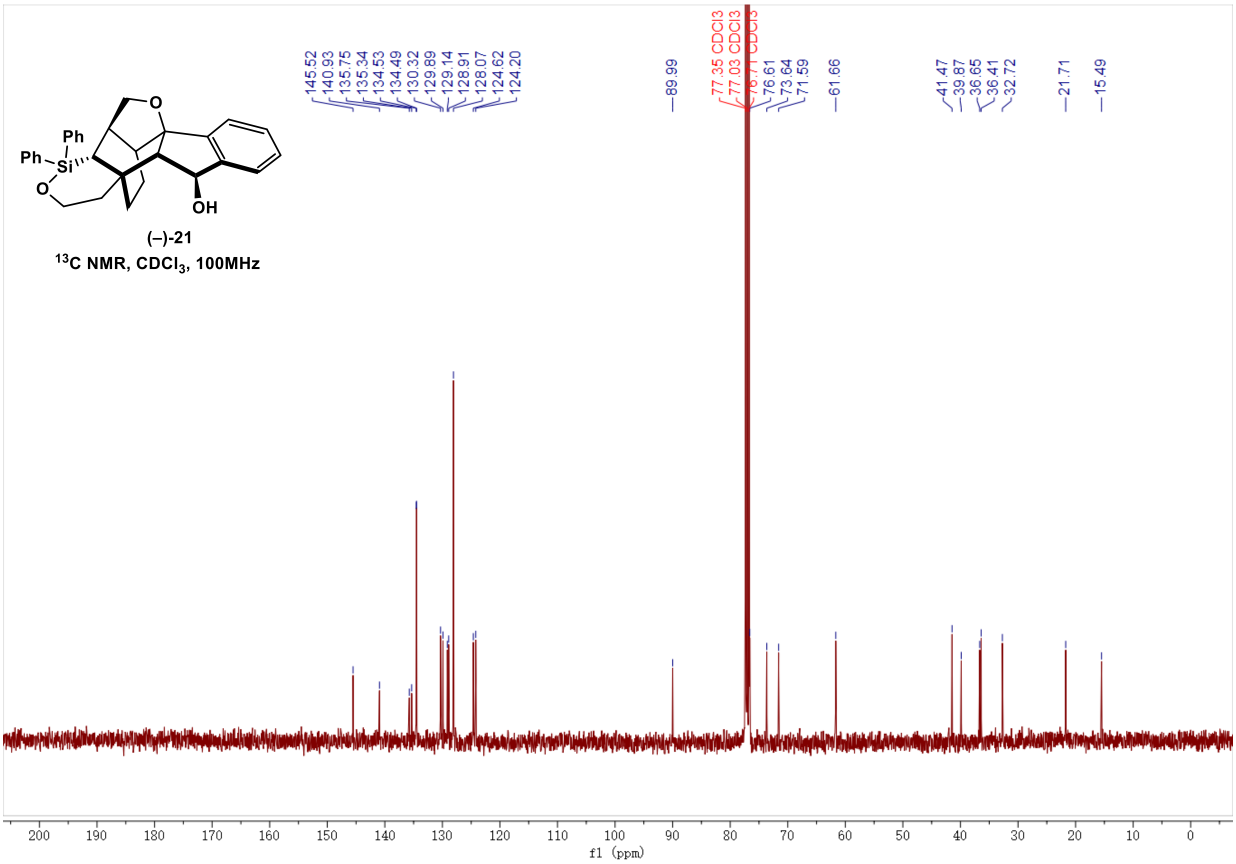

Supplementary Figure 12. NMR spectra of compound **(–)-21**

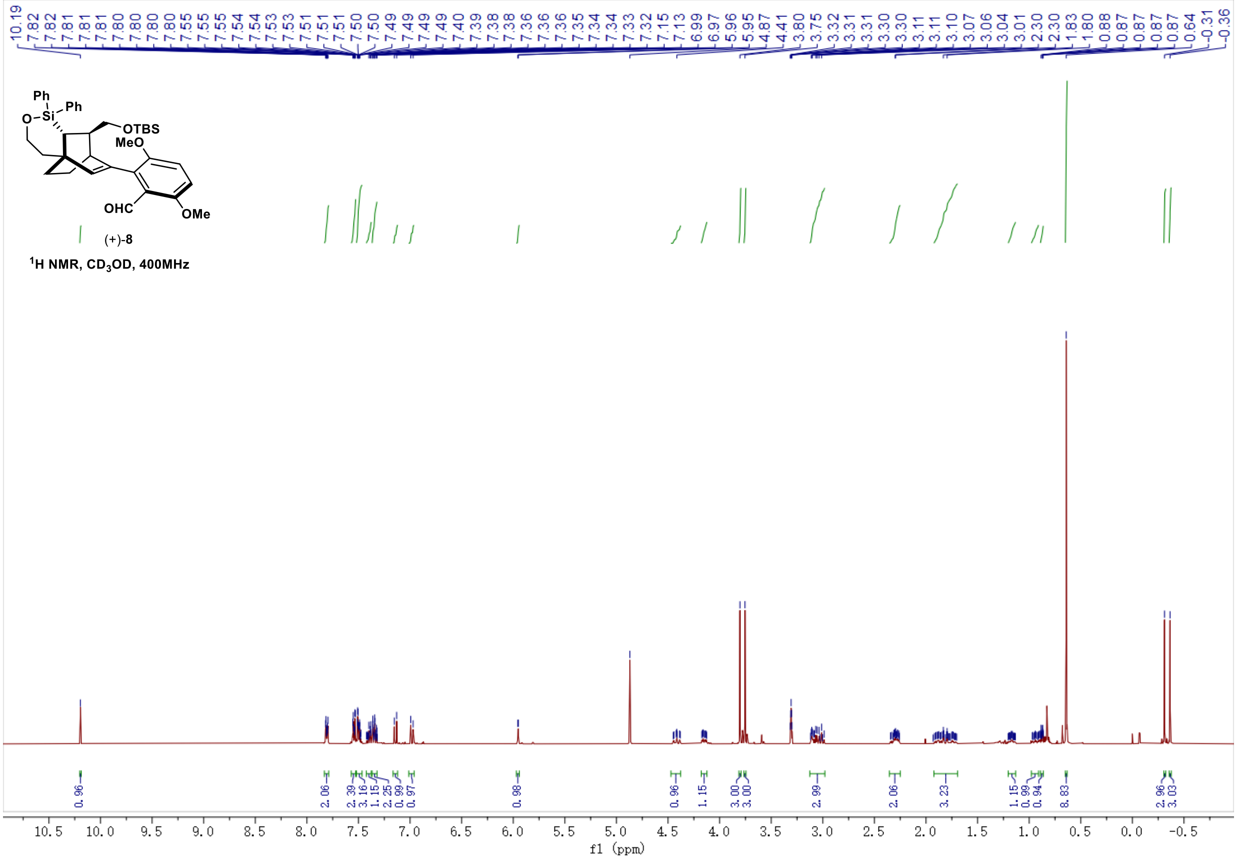

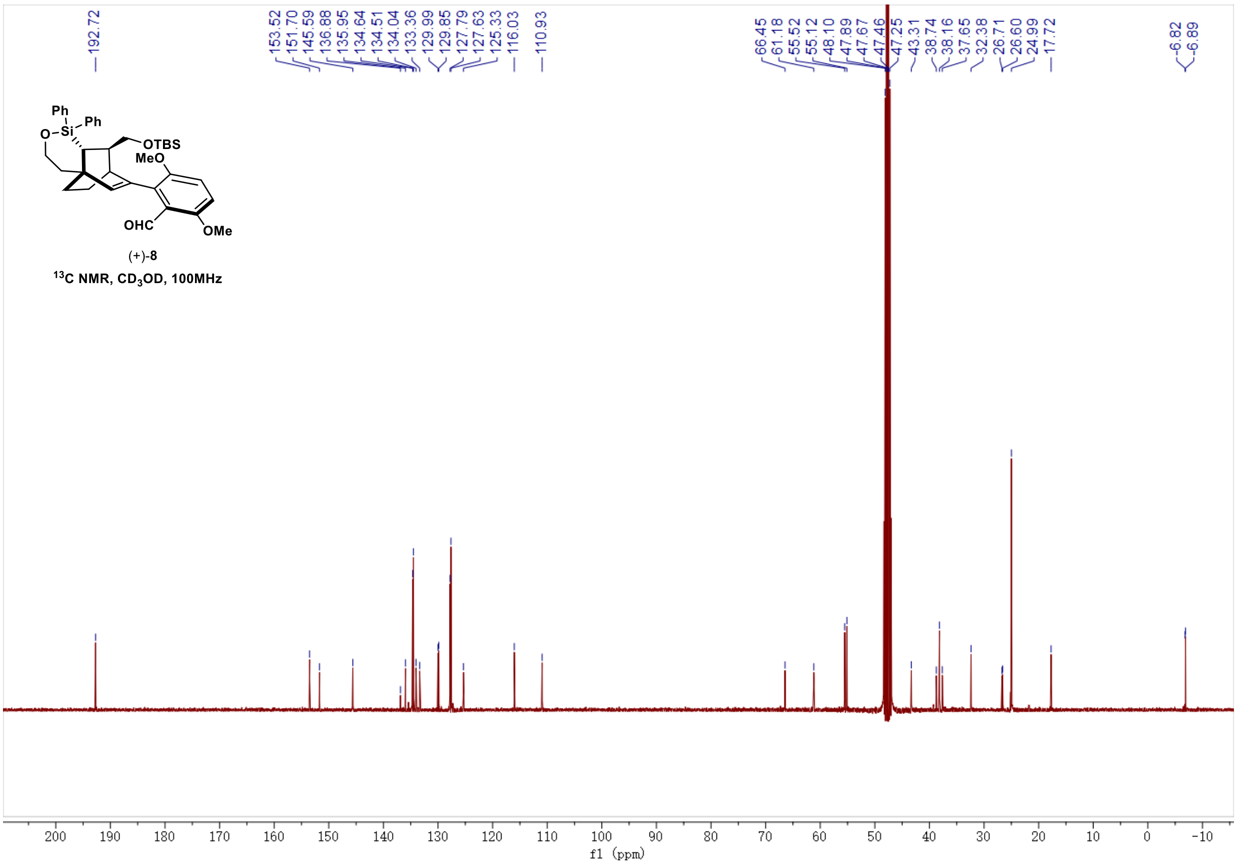

Supplementary Figure 13. NMR spectra of compound **(+)-8**

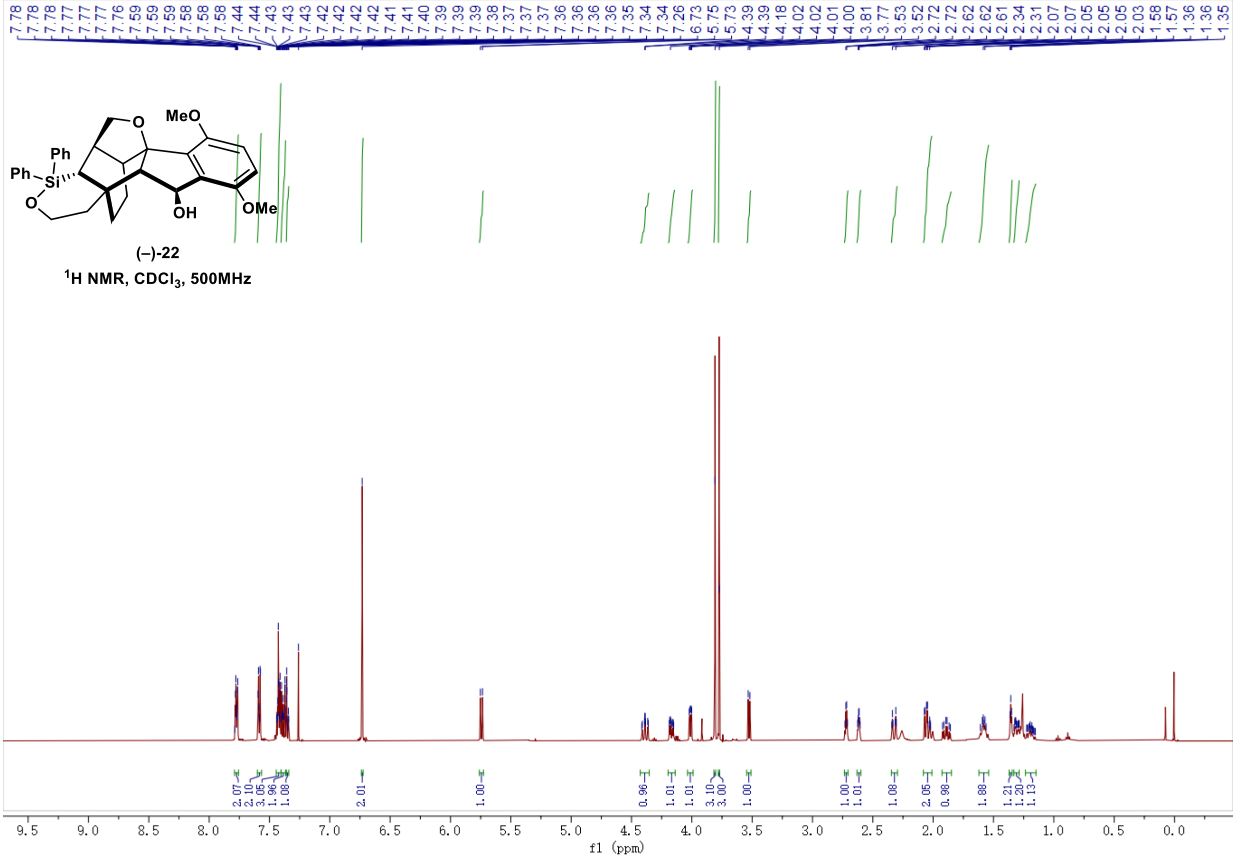

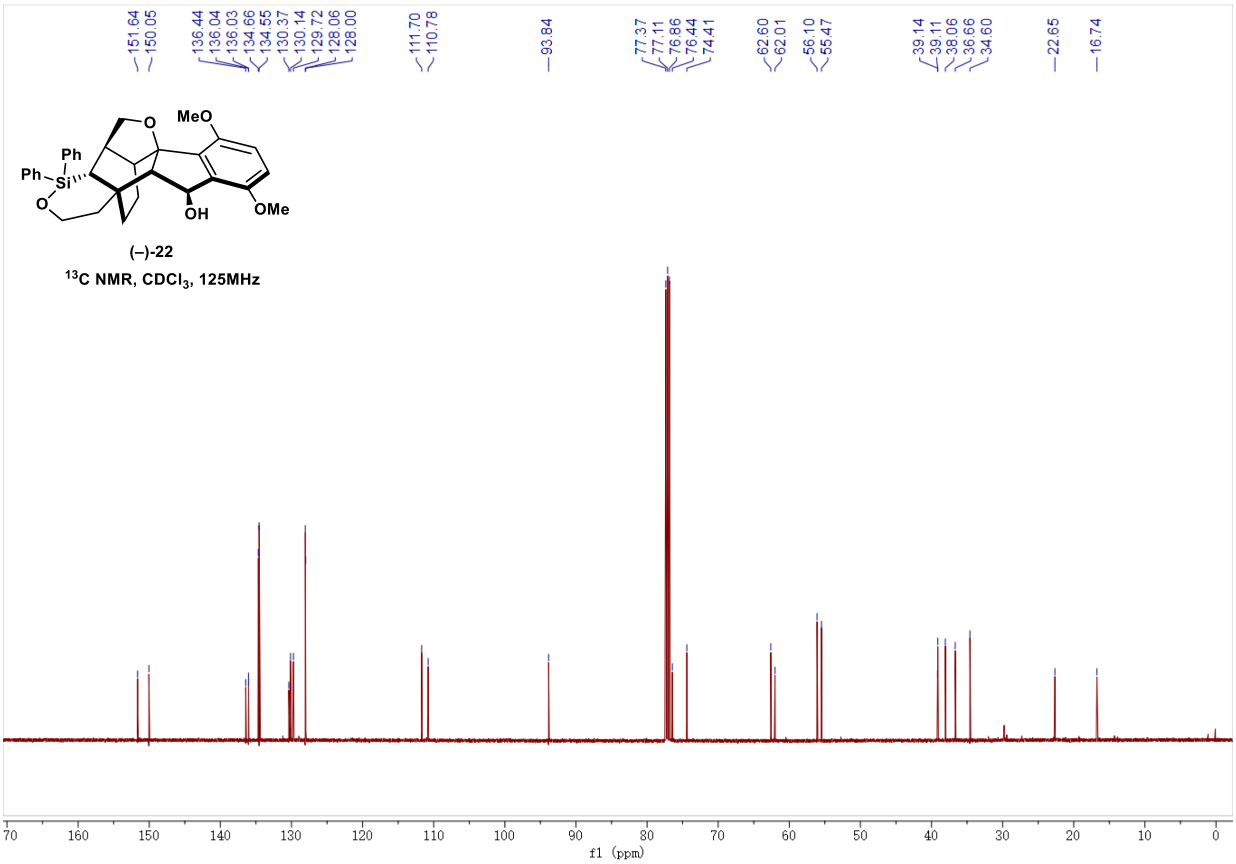

Supplementary Figure 14. NMR spectra of compound **(–)-22**

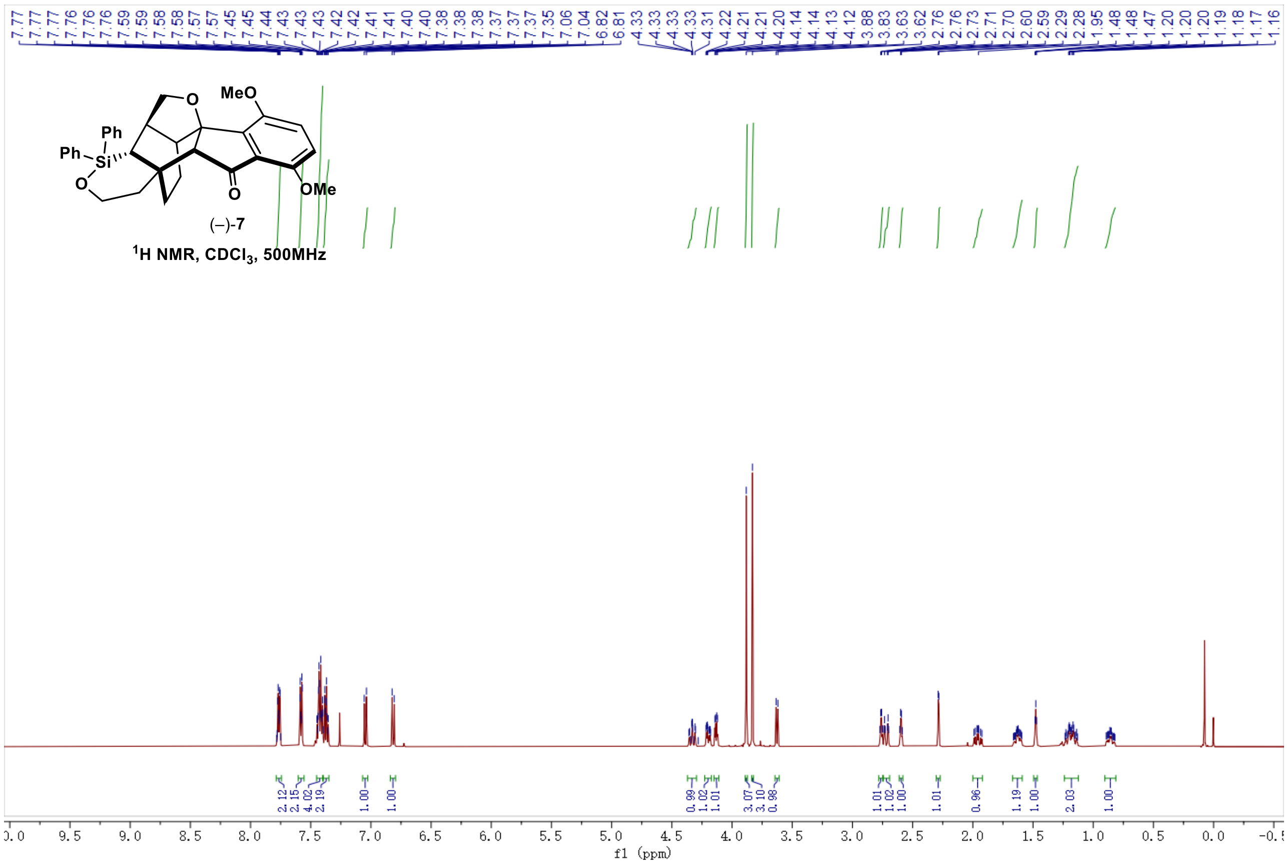

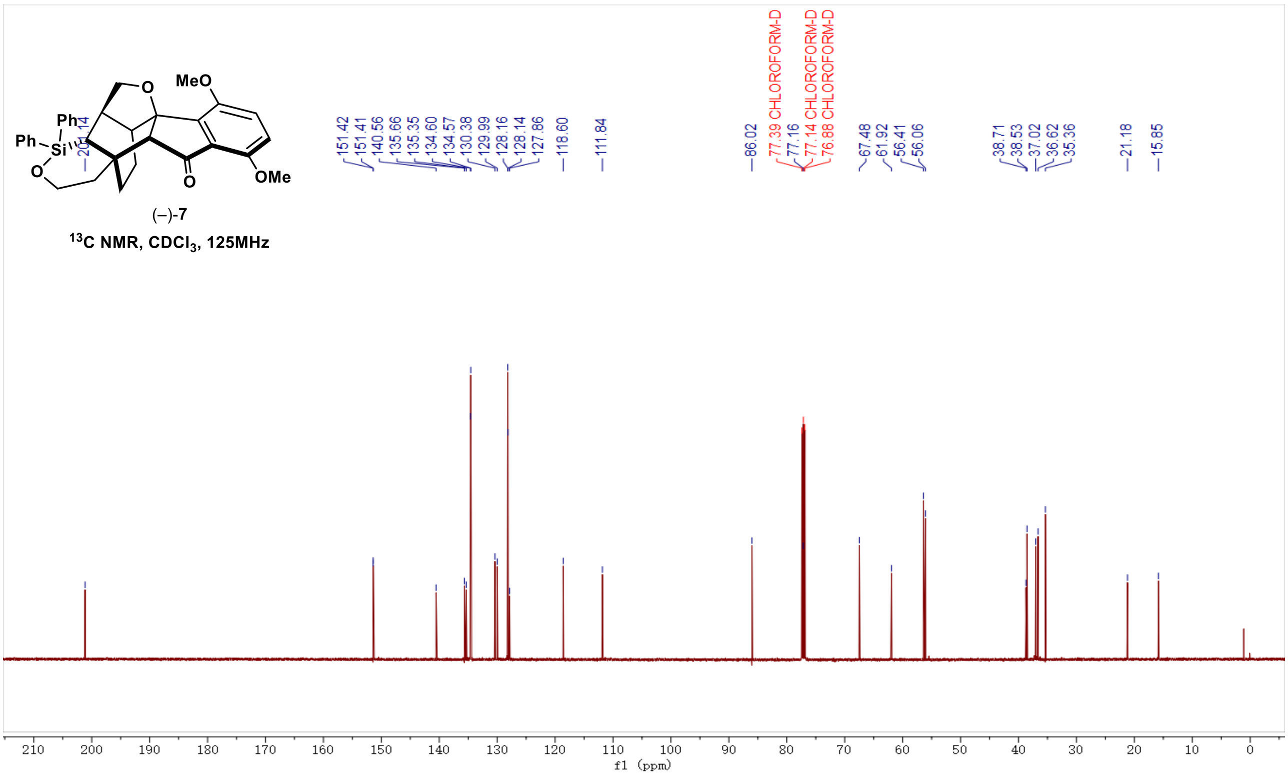

Supplementary Figure 15. NMR spectra of compound **(–)-7**

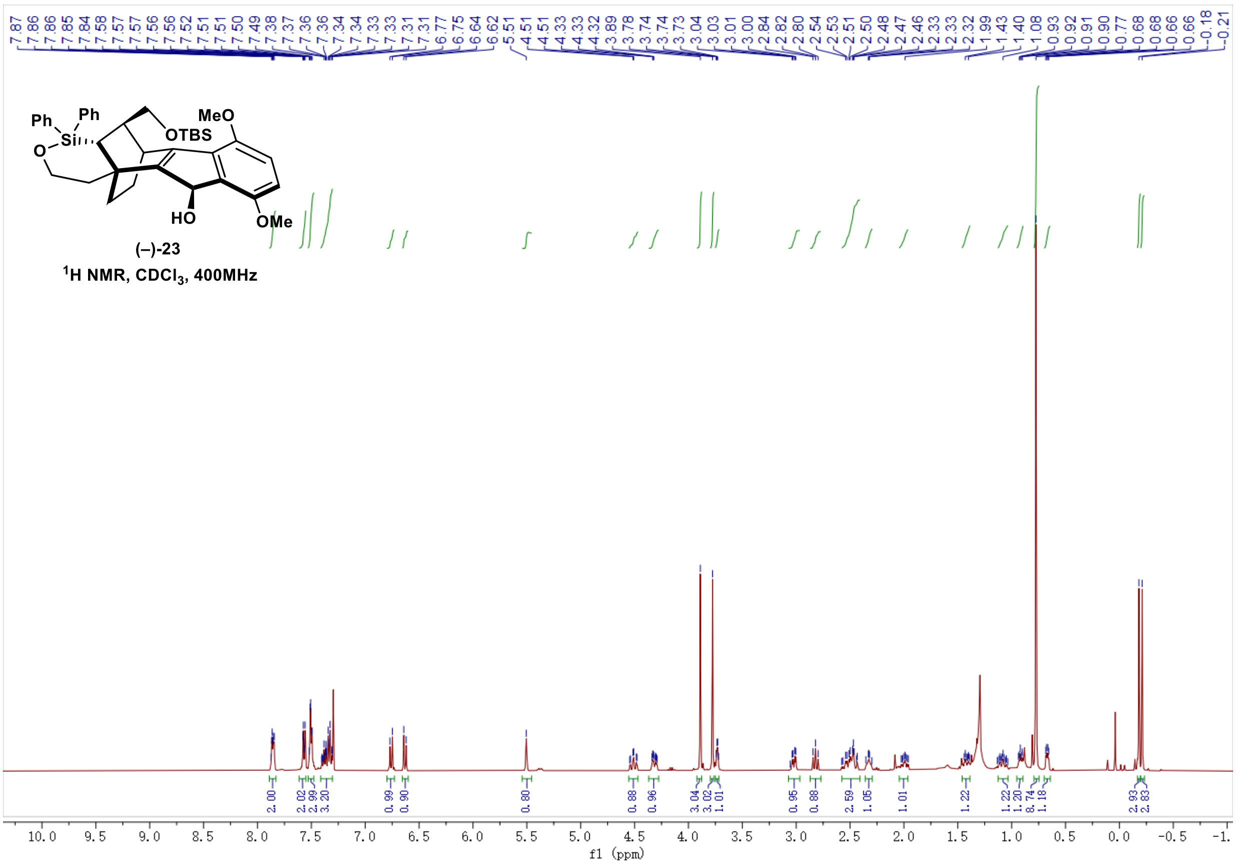

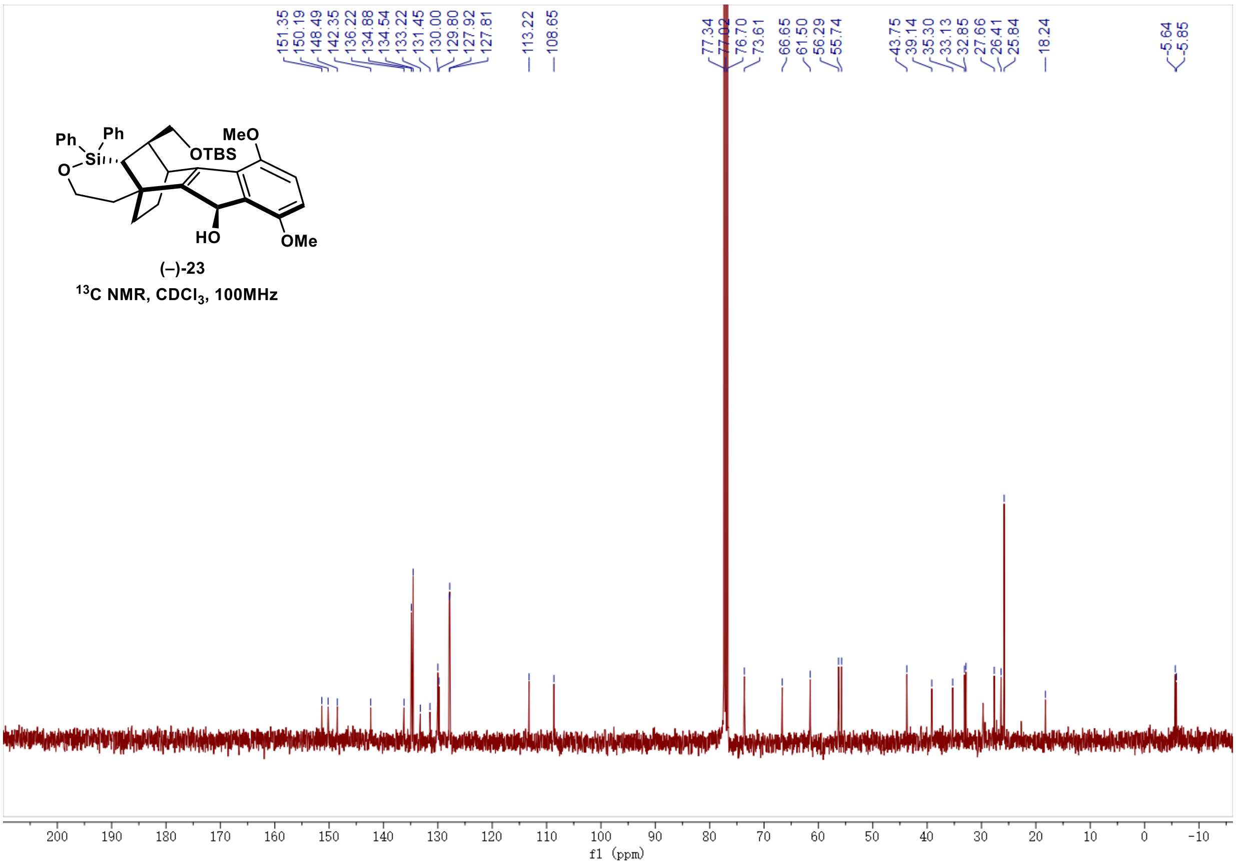

Supplementary Figure 16. NMR spectra of compound **(–)-23**

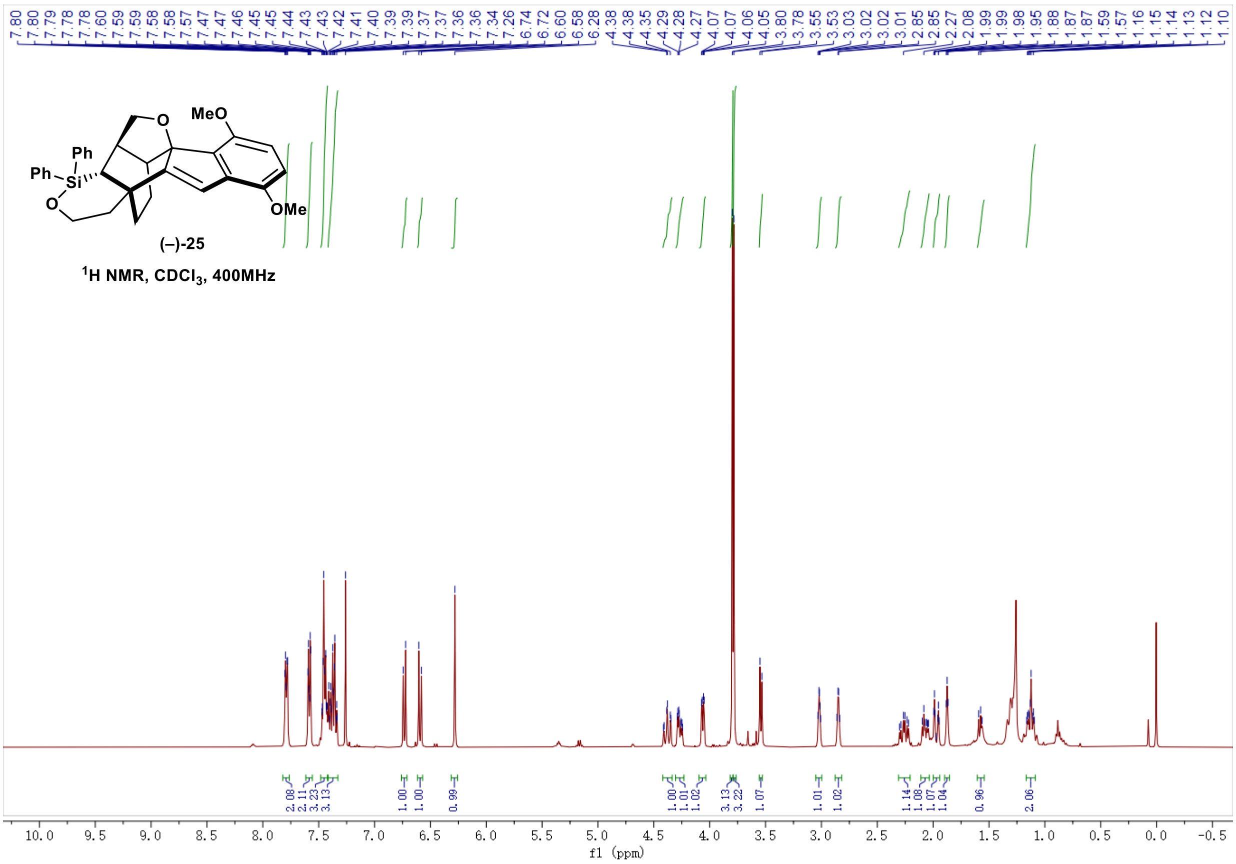

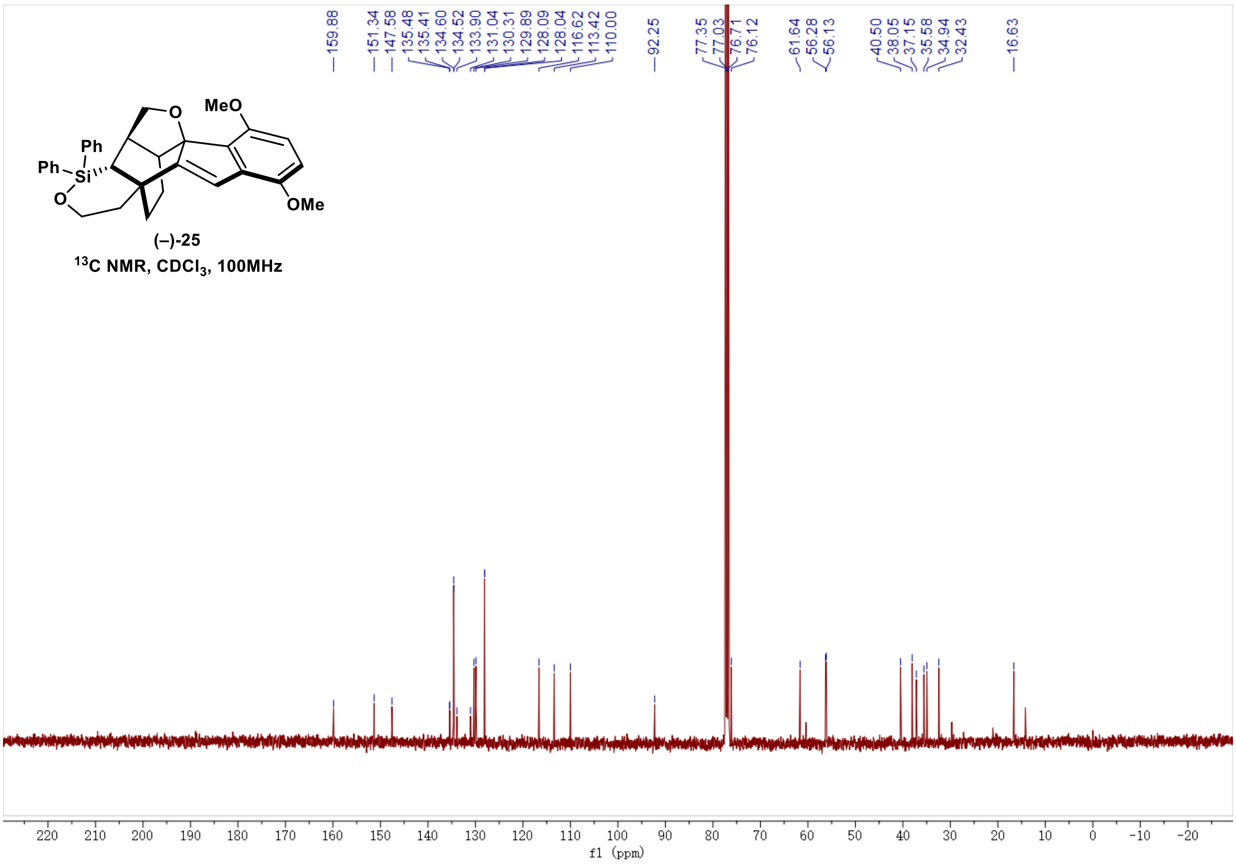

Supplementary Figure 17. NMR spectra of compound **(–)-25**

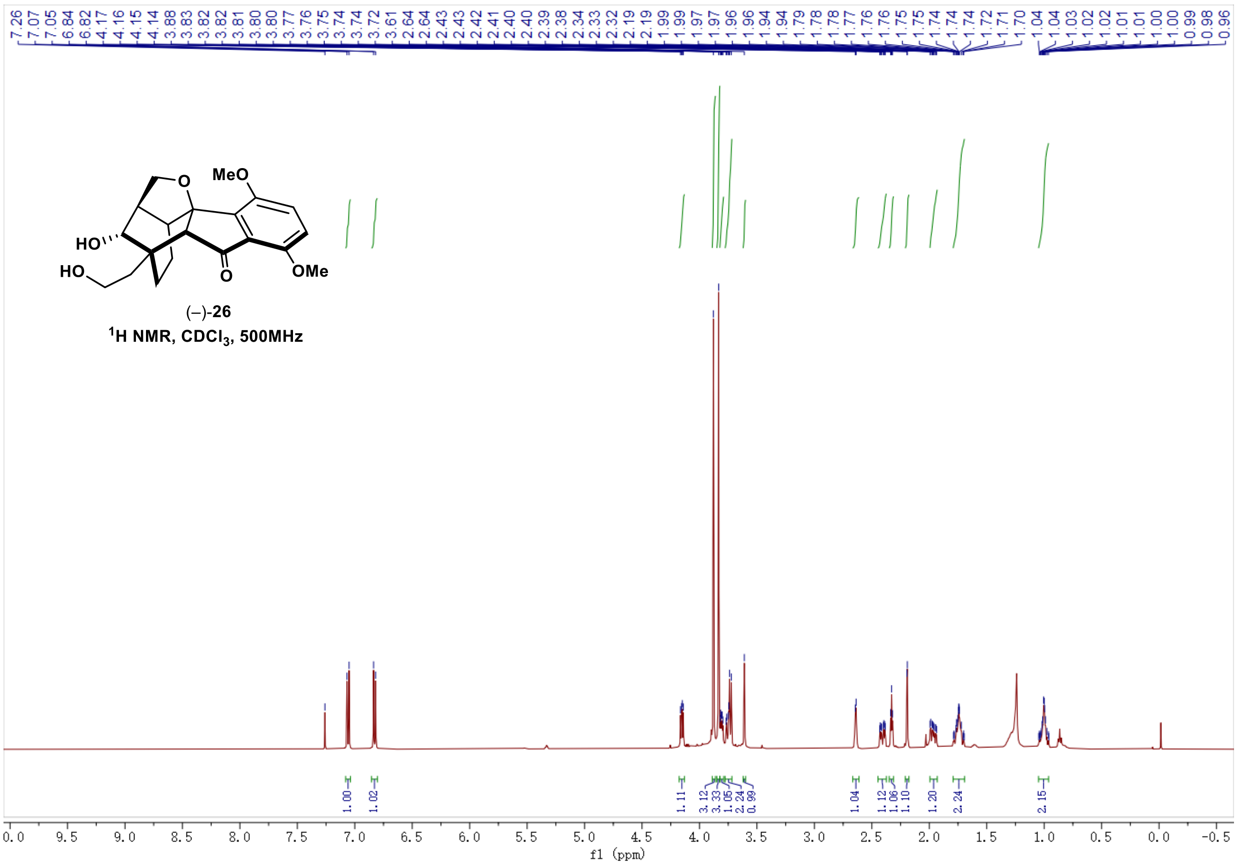

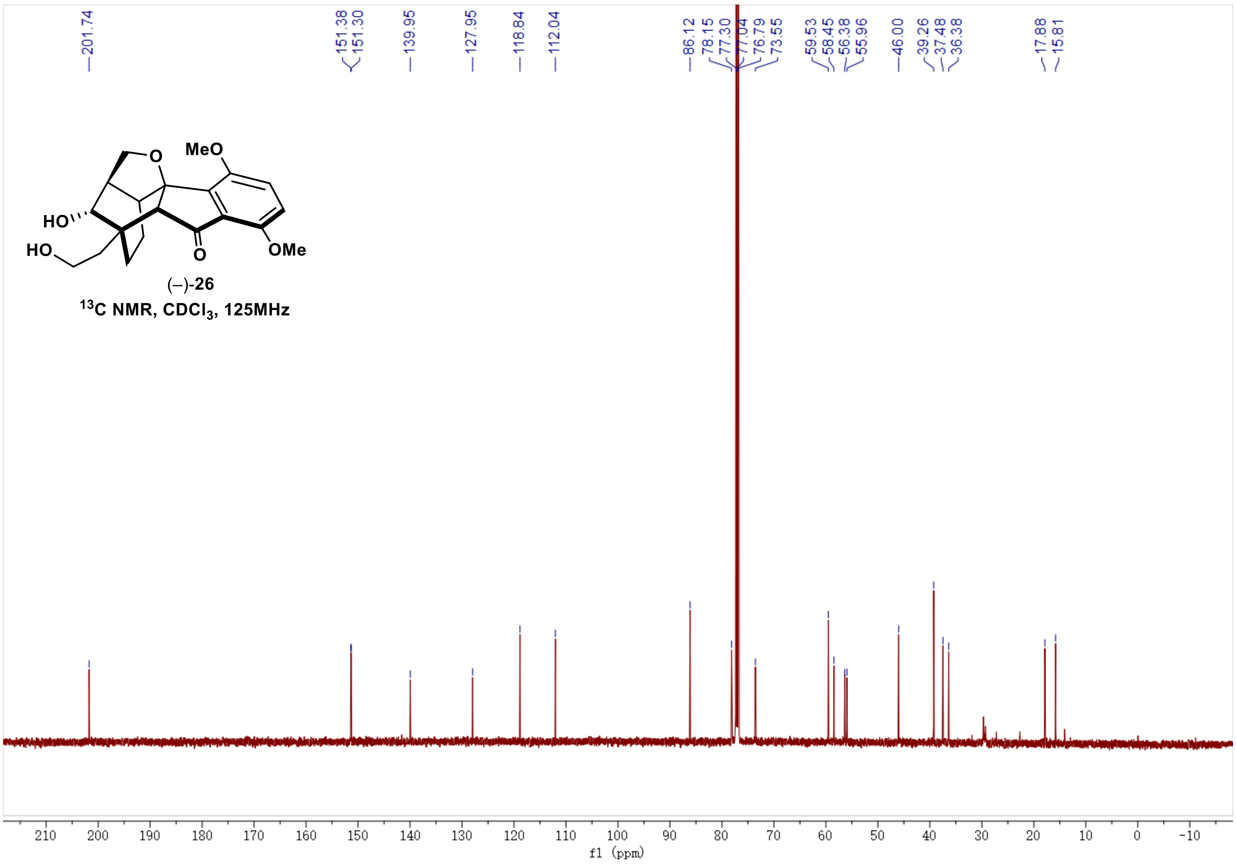

Supplementary Figure 18. NMR spectra of compound **(–)-26**

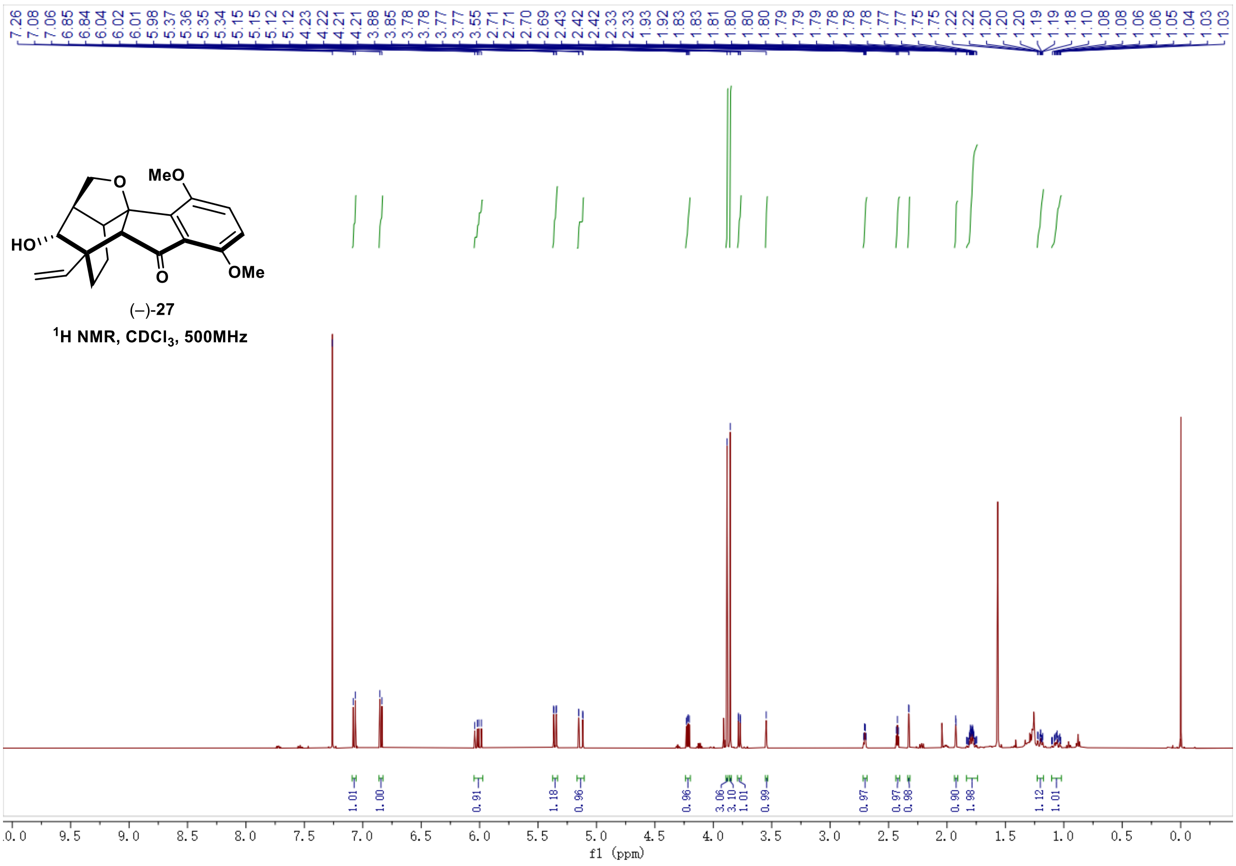

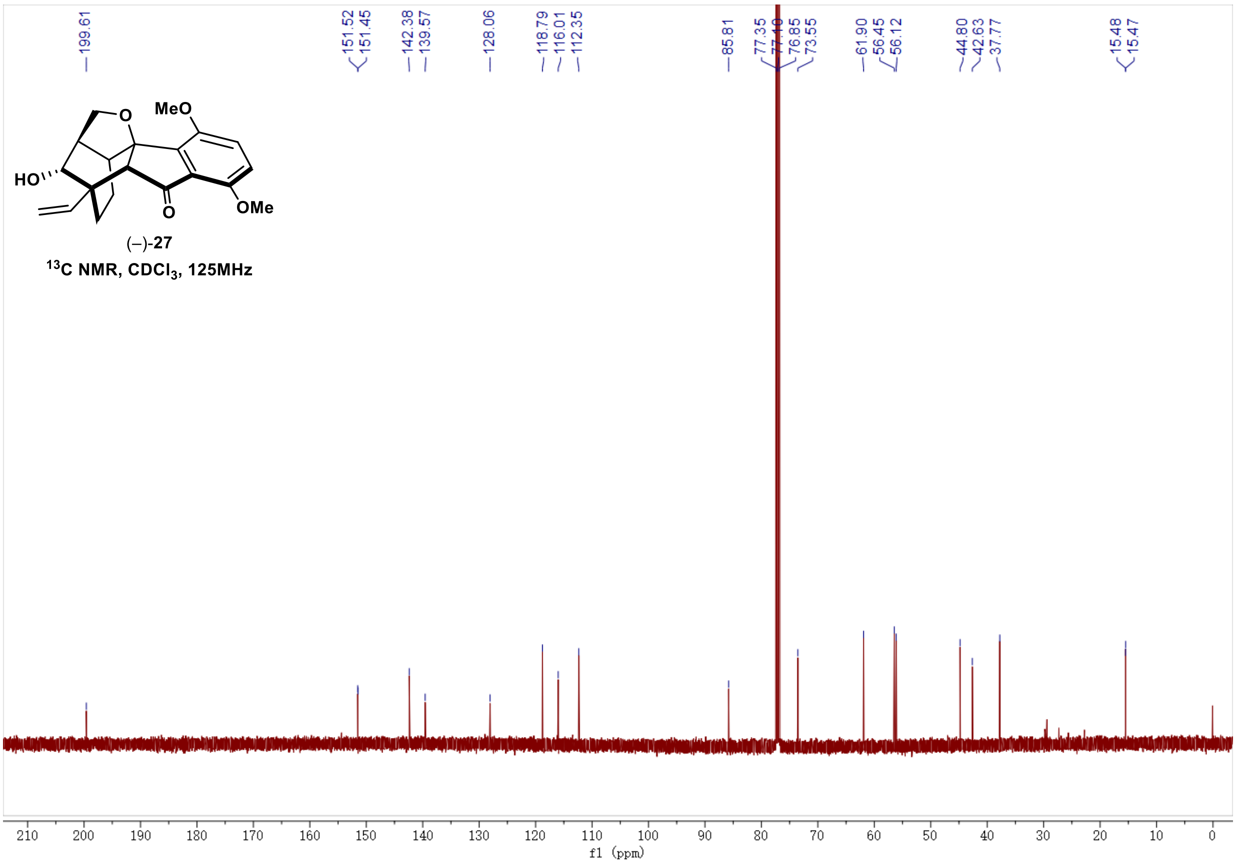

Supplementary Figure 19. NMR spectra of compound **(–)-27**

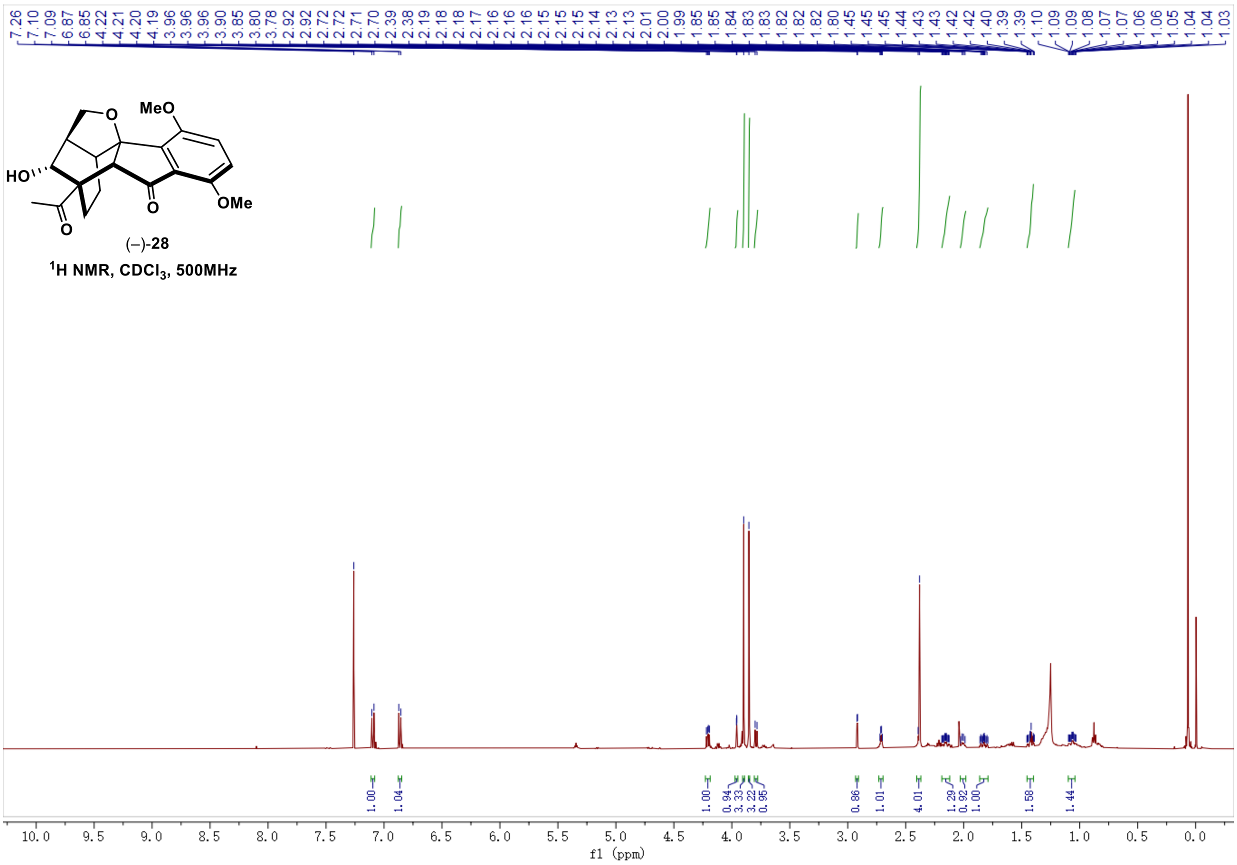

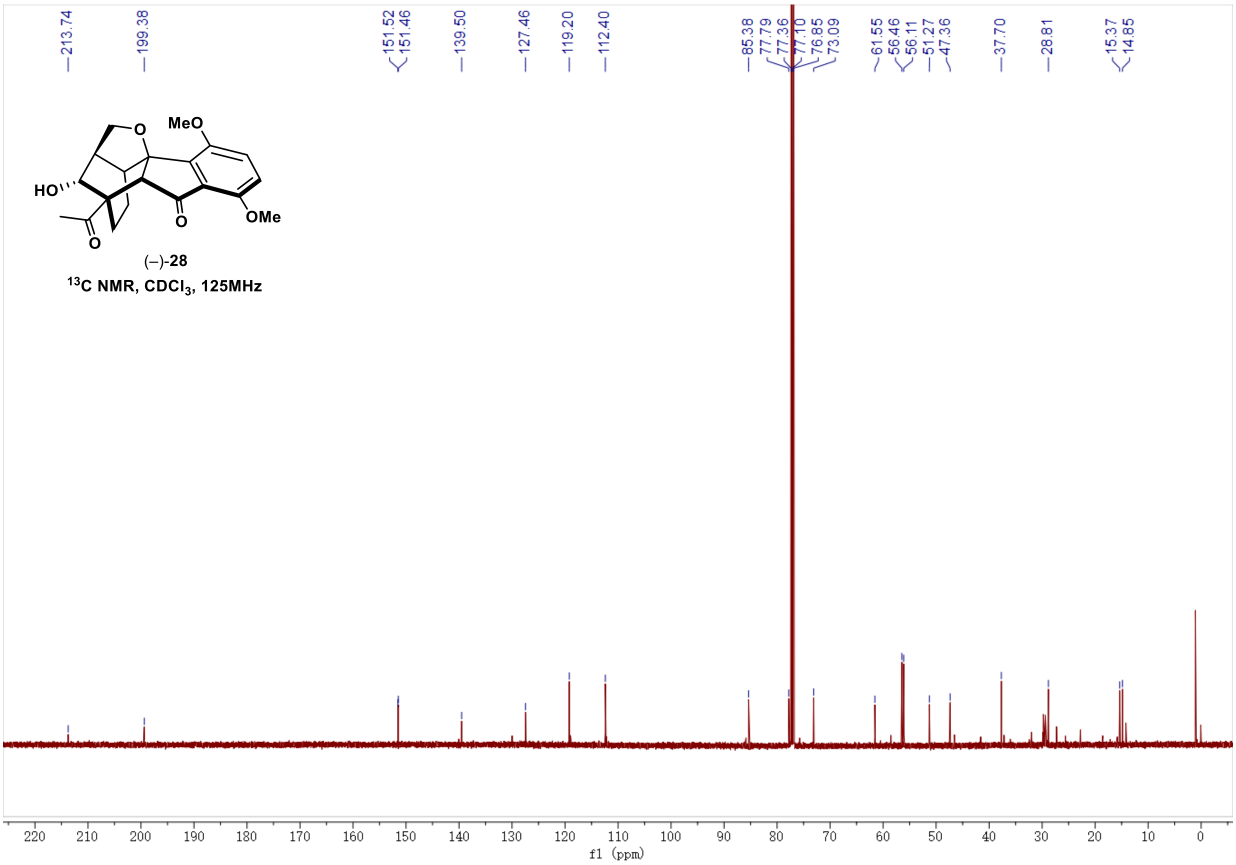

Supplementary Figure 20. NMR spectra of compound **(–)-28**

HPLC Data for Compound 11

Chiral HPLC: Chiralpak OD-H, hexane:*i*-PrOH = 80:20, 1.0 mL/min, 210 nm; tR = 11.8 min (minor), 15.9 min (major).

<Chromatogram>

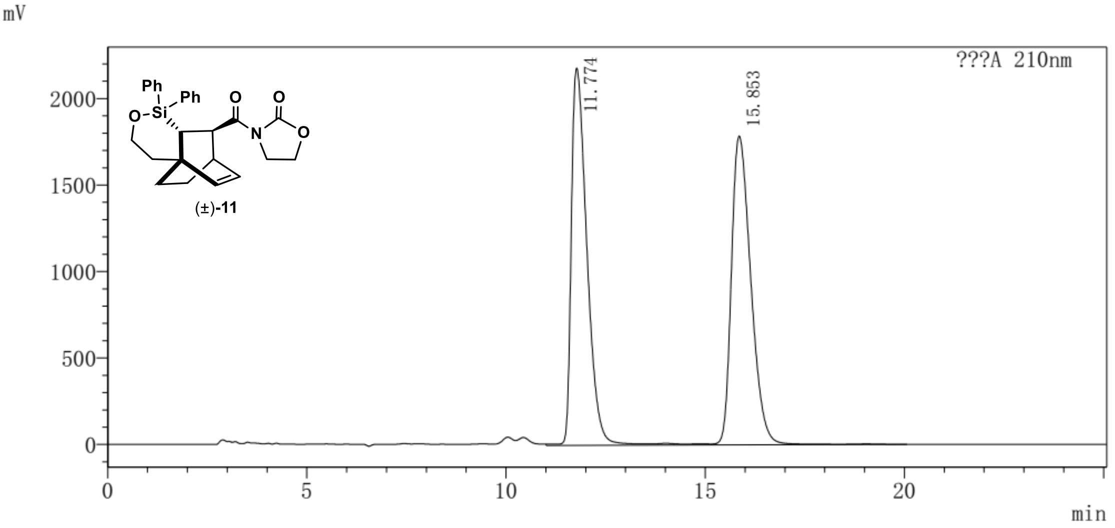

<PEAK>

???A 210nm

Peak#	Ret.Time	Area	Height	Conc.	unit	Mark	Name
1	11.774	59926205	2181271	49.729		S	
2	15.853	60580038	1785287	50.271		V	
Total		120506243	3966557				

<Chromatogram>

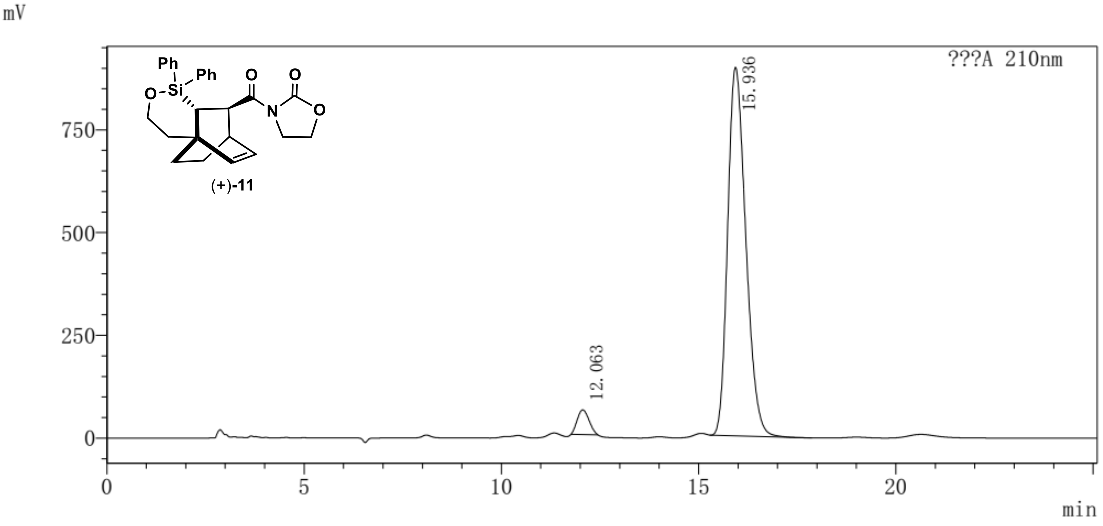

<PEAK>

???A 210nm

Peak#	Res.Time	Area	Height	Conc.	Unit	Mark	Name
1	12.063	1248994	60118	4.176		M	
2	15.936	28658805	896575	95.824		M	
Total		29907799	956694				

Supplementary Figure 21. HPLC spectra of compound **11**

3.3 Crystallographic information

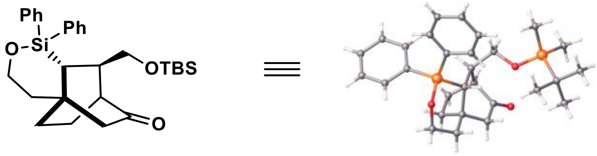

(+)-**17**

Supplementary Table 4. Crystal data and structure refinement for compound (+)-17

X-ray data for compound (+)-**17**	
CCDC number	2271397
Identification code	(+)-17
Empirical formula	C29H40O3Si2
Formula weight	492.79
Temperature/K	99.94(18)
Crystal system	orthorhombic
Space group	P212121
a/Å	11.9992(2)
b/Å	12.1536(2)
c/Å	37.7267(5)
α/°	90
β/°	90
γ/°	90
Volume/Å3	5501.82(16)
Z	8
ρcalcg/cm3	1.190
μ/mm-1	1.379
F(000)	2128.0
Crystal size/mm3	0.08 × 0.08 × 0.06
Radiation	GaKα (λ = 1.54184)
2Θ range for data collection/°	7.642 to 132.808
Index ranges	-14 ≤ h ≤ 13, -14 ≤ k ≤ 14, -44 ≤ l ≤ 44
Reflections collected	105349
Independent reflections	9612 [Rint = 0.0402, Rsigma = 0.0188]
Data/restraints/parameters	9612/308/686
Goodness-of-fit on F2	1.021
Final R indexes [I>=2σ (I)]	R1 = 0.0280, wR2 = 0.0720
Final R indexes [all data]	R1 = 0.0284, wR2 = 0.0723
Largest diff. peak/hole / e Å-3	0.24/-0.22
Flack parameter	0.000(7)

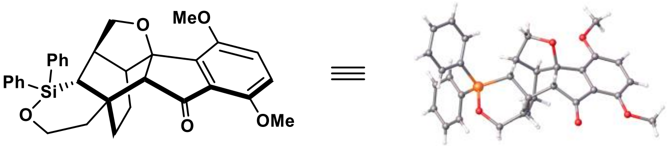

(–)-**7**

Supplementary Table 5. Crystal data and structure refinement for compound (-)-7

X-ray data for compound (-)-**7**.

CCDC number	2277937
Identification code	(-)-7
Empirical formula	C32H32O5Si
Formula weight	542.66
Temperature/K	300.00(10)
Crystal system	tetragonal
Space group	P-41212
a/Å	11.0291 (2)
b/Å	11.0291(2)
c/Å	43..2751(14)
α/°	90
β/°	90
γ/°	90
Volume/Å3	5264.0(3)
Z	8
ρcalcg/cm3	1.324
μ/mm-1	1.123
F(000)	2224.0
Crystal size/mm3	? × ? × ?
Radiation	Cu Kα (λ = 1.54184)
2Θ range for data collection/°	8.274 to 156.496
Index ranges	-11 ≤ h ≤ 13, -13 ≤ k ≤ 13, -51 ≤ l ≤ 54
Reflections collected	37426
Independent reflections	5523 [Rint = 0.0769, Rsigma = 0.0430]
Data/restraints/parameters	5523/0/346
Goodness-of-fit on F2	1.065
Final R indexes [I>=2σ (I)]	R1 = 0.0510, wR2 = 0.1193
Final R indexes [all data]	R1 = 0.0857, wR2 = 0.1532
Largest diff. peak/hole / e Å-3	0.25/-0.20
Flack parameter	0.03(2)

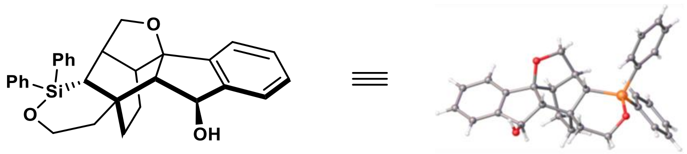

(±)-21

Supplementary Table 6. Crystal data and structure refinement for compound (±)-21

X-ray data for compound (±)-**21**	
CCDC number	2312931
Identification code	(±)-21
Empirical formula	C30H30O3Si
Formula weight	466.63
Temperature/K	100.00(10)
Crystal system	triclinic
Space group	P-1
a/Å	8.85330(10)
b/Å	10.96160(10)
c/Å	12.95210(10)
α/°	104.9690(10)
β/°	93.0220(10)
γ/°	93.4100(10)
Volume/Å3	1209.13(2)
Z	2
ρcalcg/cm3	1.282
μ/mm-1	1.091
F(000)	496.0
Crystal size/mm3	0.42 × 0.25 × 0.2
Radiation	Cu Kα (λ = 1.54184)
2Θ range for data collection/°	7.082 to 153.416
Index ranges	-11 ≤ h ≤ 8, -13 ≤ k ≤ 13, -16 ≤ l ≤ 16
Reflections collected	20605
Independent reflections	4873 [Rint = 0.0215, Rsigma = 0.0128]
Data/restraints/parameters	4873/0/308
Goodness-of-fit on F2	1.056
Final R indexes [I>=2σ (I)]	R1 = 0.0362, wR2 = 0.0938
Final R indexes [all data]	R1 = 0.0368, wR2 = 0.0943
Largest diff. peak/hole / e Å-3	0.25/-0.43

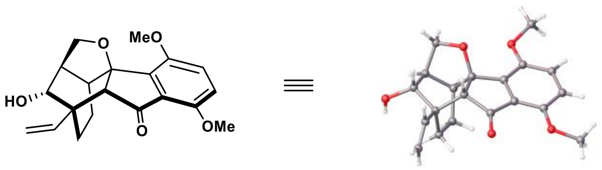

(−)-**27**

Supplementary Table 7. Crystal data and structure refinement for compound (-)-27

X-ray data for compound (-)-**27**	
CCDC number	2284054
Identification code	(-)-27
Empirical formula	C20H22O5
Formula weight	342.37
Temperature/K	100.01(10)
Crystal system	orthorhombic
Space group	P-212121
a/Å	6.75558(5)
b/Å	12.42947(9)
c/Å	19.93730(14)
α/°	90
β/°	90
γ/°	90
Volume/Å3	1674.10(2)
Z	4
ρcalcg/cm3	1.358
μ/mm-1	0.796
F(000)	728.0
Crystal size/mm3	0.3 × 0.25 × 0.22
Radiation	Cu Kα (λ = 1.54184)
2Θ range for data collection/°	8.382 to 152.738
Index ranges	-8 ≤ h ≤ 8, -15 ≤ k ≤ 13, -25 ≤ l ≤ 25
Reflections collected	14137
Independent reflections	3366 [Rint = 0.0157, Rsigma = 0.0108]
Data/restraints/parameters	3366/0/230
Goodness-of-fit on F2	1.024
Final R indexes [I>=2σ (I)]	R1 = 0.0241, wR2 = 0.0625
Final R indexes [all data]	R1 = 0.0243, wR2 = 0.0626
Largest diff. peak/hole / e Å-3	0.19/-0.14
Flack parameter	0.02(3)

4. Supplementary References

[1] Takeshita, T., Seki, Y., Kawamoto, K., Murai, S. & Sonoda. N. The catalyzed reaction of .alpha.,.beta.-unsaturated esters with various hydrosilanes. *J. Org. Chem.* **52**, 4864-4868 (1987)

[2] Shvartsbart, A. & Smith III, A. B. Total Synthesis of (−)-Calyciphylline N. *J. Am. Chem. Soc*. **136**, 870−873 (2014).

[3] Sieburth, S. M. & Lang, J. A practical synthesis of difunctional organosilane reagents and their application to the Diels−Alder reaction. *J. Org. Chem*. **64**, 1780−1781 (1999).
